# An important role of _L_-fucose biosynthesis and protein fucosylation genes in Arabidopsis immunity

**DOI:** 10.1111/nph.15639

**Published:** 2019-01-23

**Authors:** Li Zhang, Bradley C. Paasch, Jin Chen, Brad Day, Sheng Yang He

**Affiliations:** 1 Department of Energy Plant Research Laboratory, East Lansing, MI 48824, USA; 2 Howard Hughes Medical Institute, Michigan State University, East Lansing, MI 48824, USA; 3 Department of Plant Biology, Michigan State University, East Lansing, MI 48824, USA; 4 Department of Biochemistry and Molecular Biology, Michigan State University, East Lansing, MI 48824, USA; 5 Department of Computer Science and Engineering, Michigan State University, East Lansing, MI 48824, USA; 6 Department of Plant, Soil and Microbial Sciences, Michigan State University, East Lansing, Michigan 48824; 7 Plant Resilience Institute, Michigan State University, East Lansing, MI 48824, USA

**Keywords:** coronatine, fucosylation, jasmonate, _L_-fucose, plant immunity, stomata

## Abstract

Plants mount coordinated immune responses to defend against pathogens. However, the cellular components required for plant immunity are not fully understood. The jasmonate-mimicking coronatine (COR) toxin produced by *Pseudomonas syringae* pv. *tomato* (*Pst*) DC3000 functions to overcome plant immunity. We previously isolated eight Arabidopsis (*scord*) mutants that exhibit increased susceptibility to a COR-deficient mutant of *Pst* DC3000. Among them, the *scord6* mutant exhibits defects both in stomatal closure response and in restricting bacterial multiplication inside the apoplast. However, the identity of *SCORD6* remained elusive.

In this study, we aim to identify the *SCORD6* gene.

We identified *SCORD6* via next-generation sequencing and found it to be *MURUS1* (*MUR1*), which is involved in the biosynthesis of GDP-_L_-fucose.

Discovery of *SCORD6* as *MUR1* led to a series of experiments that revealed a multi-faceted role of _L_-fucose biosynthesis in stomatal and apoplastic defenses as well as in pattern-triggered immunity and effector-triggered immunity, including glycosylation of pattern-recognition receptors. Furthermore, compromised stomatal and/or apoplastic defenses were observed in mutants of several fucosyltransferases with specific substrates (e.g., *O*-glycan, *N*-glycan or the DELLA transcriptional repressors). Collectively, these results uncover a novel and broad role of _L_-fucose and protein fucosylation in plant immuinity.

## Introduction

In nature, plants are exposed to a wide variety of microbes, including pathogens. To protect against pathogen attacks, plants have developed various defense strategies, including preformed physical barriers (e.g., cuticles and cell walls) and antimicrobial compounds, as well as an inducible innate immune system (Jones & Dangl 2006; Bigeard et al. 2015). Research on Arabidopsis immune responses suggests that the inducible plant innate immune system consists of two signaling branches. The first branch involves recognition of conserved pathogen/microbe-associated molecular patterns (PAMPs/MAMPs) by pattern-recognition receptors (PRRs), resulting in pattern-triggered immunity (PTI) (Macho & Zipfel 2014). For example, perception of bacterial flagellin or a highly conserved 22 amino acid epitope, flg22, is mediated by the FLAGELLIN-SENSITIVE2 (FLS2) receptor (Gomez-Gomez & Boller 2000; Chinchilla et al. 2006). Similarly, elongation factor-Tu (EF-Tu) or a 26-amino acid epitope of EF-Tu, elf26, is recognized by the EF-Tu receptor (EFR) (Kunze et al. 2004; Zipfel et al. 2006). The second branch of inducible innate immunity, designated effector-triggered immunity (ETI), is triggered by direct or indirect recognition of pathogen virulence proteins (“effectors”) by cognate disease resistance (R) proteins (Cui et al. 2015). For example, the effector protein AvrRpt2 from the bacterial pathogen *Pseudomonas syringae* pv. *tomato* (*Pst*) is indirectly perceived by the RESISTANCE TO *P. syringae* 2 (RPS2) protein and activates downstream ETI signaling and defense outputs, including the hypersensitive cell death response (Axtell & Staskawicz 2003; Mackey et al. 2003).

As foliar pathogens, strains of *P. syringae* often go through an initial epiphytic phase on the surfaces of the above-ground parts (collectively called the phyllosphere) of plants and a subsequent endophytic phase inside the apoplast (intercellular spaces between mesophyll cells), where aggressive multiplication takes place (Melotto et al. 2008; Xin et al. 2018). The transition from the epiphytic phase to endophytic phase involves the entry of *P. syringae* into the plant apoplast via wounds or natural openings, such as stomata, in the epidermis (Hirano & Upper 2000). In response, plants have evolved elaborate defense responses to counter bacteria during invasion through stomata and inside the apoplast. As the first line of defense, plants respond to live bacteria or MAMPs (such as flg22 and elf26) by closing stomatal aperture (Melotto et al. 2006; Arnaud & Hwang 2015; Ye & Murata 2016). Specific PRRs are necessary for plants to sense MAMPs and induce stomatal closure (Melotto et al. 2006; Zhang et al. 2008; Mersmann et al. 2010). In addition, salicylic acid (SA) biosynthesis and signaling are also required for pathogen-induced stomatal closure (Arnaud & Hwang 2015; Ye & Murata 2016; Melotto et al. 2017). Moreover, both MAMP perception/ signaling and SA biosynthesis/signaling are also important for plant defense against bacterial multiplication inside the apoplast (Heese et al. 2007; Genger et al. 2008; Nekrasov et al. 2009; Boutrot et al. 2010; Zeng & He 2010; Zeng et al. 2011; Uddin et al. 2017).

The phytotoxin coronatine (COR) plays multiple roles in *Pst* DC3000 infection of Arabidopsis and is implicated in overcoming both stomatal and apoplastic defenses to facilitate bacterial invasion and multiplication, respectively (Zeng et al. 2011). COR is a remarkable structural mimic of the active form of plant hormone jasmonoyl-isoleucine (JA-Ile), and its virulence roles appear to be mediated through activating jasmonate (JA) signaling (Zhang et al. 2017) and other mechanisms (Kim et al. 2017). COR interferes with MAMP-induced stomatal closure and triggers stomatal reopening (Melotto et al. 2006; Zheng et al. 2012; Montillet et al. 2013; Melotto et al. 2017). COR also suppresses ABA-, oxylipin- and dark-induced stomatal closure (Melotto et al. 2006; Montillet et al. 2013; Panchal et al. 2016). In addition, COR is required for *Pst* DC3000 multiplication in the apoplast, as demonstrated by infiltration-based inoculation that delivers bacteria directly into the apoplast, bypassing stomatal defense (Brooks et al. 2004; Zeng & He 2010). Importantly, research indicates that COR suppresses stomatal closure and apoplastic defense through antagonizing SA signaling (Brooks et al. 2005; Melotto et al. 2006; Zeng & He 2010; Zheng et al. 2012).

The multiple roles of COR in *Pst* DC3000 infection was also demonstrated in an unbiased genetic screen by Zeng and colleagues for Arabidopsis mutants with increased *s**usceptibility to* *CO**R-**d**eficient Pst* DC3000 (*scord*) (Zeng et al. 2011). Of the eight *scord* mutants, two were affected only in stomatal response, two were affected only in apoplastic defense, and the other four, including *scord6*, were affected in both. In this study, we identified the *SCORD6* gene*. SCORD6* encodes an isoform of GDP-_D_-mannose-4,6-dehydratase (GMD), GMD2 (also named *MURUS1* [*MUR1*]; Reiter et al. 1993), which has been shown to be involved in the *de novo* synthesis of GDP-_L_-fucose, but has not been reported to play a role in plant-bacterial interactions. We found that a defect in _L_-fucose synthesis has a broad effect on multiple aspects of plant defense, including stomatal defense, apoplastic defense, PTI and ETI. Further analysis of known alleles of *SCORD6* as well as mutants of specific fucosyltransferases showed a requirement of intact *N*-glycans, *O*-glycans and mono-*O*-fucosylated proteins for proper plant immune responses. Overall, the identification of the *SCORD6* gene highlights an important and previously underappreciated role of _L_-fucose biosynthesis and fucosylation of polysaccharides and proteins in multiple immune responses in Arabidopsis.

## Materials and methods

### Plant materials and growth condition

Arabidopsis mutants were obtained from the Arabidopsis Biological Resource Center (Alonso et al. 2003), including *mur1-1* (CS6243) (Reiter et al. 1993; Bonin et al. 1997), *mur2-1* (CS8565) (Reiter et al. 1997; Vanzin et al. 2002), *fut4* (SAIL_284_B05) (Liang et al. 2013), *fut6* (SALK_099500) (Liang et al. 2013), *cgl1-3* (SALK_073650) (Frank et al. 2008), *fucTa* (SALK_087481) (Strasser et al. 2004), *fucTb* (SALK_063355) (Strasser et al. 2004), *fut13-2* (SALK_067444) (Anderson et al. 2012), *stt3a-2* (SALK_058814) (Koiwa et al. 2003), *stt3b-1* (SALK_033391) (Koiwa et al. 2003), *spy-3* (CS6268) (Jacobsen & Olszewski 1993) and *spy-5* (CS8094) (Wilson & Somerville 1995). Arabidopsis plants used for stomatal closure assays and bacterial infection assays were grown under 12-h light/12-h dark photoperiod at 22-23 °C, 100 μmol m^−2^ s^−1^ and ~50% humidity for 4 to 5 weeks.

### Identification of the *SCORD6* gene

Genomic DNA of the *scord6* mutant and the parental Col-7 plant was extracted using PowerPlant Pro DNA Isolation Kit (Mo-Bio) and sent to the Michigan State University (MSU) Research Technology Support Facility (RTSF) Genomic Core for paired-end sequencing using Illumina’s HiSeq 2500 next-generation sequencer. A total of ~3 Gbp genome sequences for each sample were obtained and the coverage was 21- and 26-fold for the *scord6* mutant and Col-7 wild-type, respectively. The read quality control was assessed using FastQC (Andrew 2010). The reads of Col-7 and the *scord6* mutant were assembled based on Col-0 TAIR10 genome sequence and the sequence of the activation-tagging vector pSKI015 (Weigel et al. 2000) using Bowtie2 (Langmead & Salzberg 2012). Sequence variations including SNPs and INDELs were identified using SAMtools and VCFtools (H. Li et al. 2009; Danecek et al. 2011). Homozygous variations (QUAL value >= 30) detected only in the *scord6* genome, but not in the parental Col-7 genome, were selected and mapped back to the previously identified candidate regions by physical mapping (Zeng et al. 2011). Only nonsynonymous SNPs or INDELs located within the open-reading frame of a gene in the candidate regions of the *scord6* genome were selected and confirmed by Integrative Genomics Viewer (IGV) (Robinson et al. 2011) and PCR. Primers used in targeted PCR for confirming the presence of deletion in *scord6* mutant were described in Supplemental Table 1.

### Generation of transgenic Arabidopsis

The coding DNA sequence (CDS) of the *MUR1* (AT3G51160) gene was amplified from a total mRNA extract of Col-0 leaf tissue and cloned into the donor vector pDONR207 via BP Clonase II (Invitrogen), generating the entry clone. The entry clone was then recombined with the destination vector pGWB517 (Nakagawa et al. 2007) using LR Clonase II (Invitrogen) to create *35S*_*pro*_*:MUR1-4xMyc*. The confirmed construct was introduced into *Agrobacterium tumefaciens* (GV3101) by electroporation. *35S*_*pro*_*:MUR1-4xMyc* construct was then transformed into the *scord6* mutant via *A. tumefaciens*-mediated Arabidopsis transformation (Clough & Bent 1998). Half-strength Murashige and Skoog (MS) medium with 50 μg ml^−1^ hygromycin was used to select transgenic seedlings expressing the *35S*_*pro*_*:MUR1-4xMyc* transgene.

### Bacterial infection assays

*P. syringae* infection assays in Arabidopsis were performed as described previously (Yao et al. 2013). Briefly, four- to five-week-old Arabidopsis plants were dip-inoculated with bacterial suspension (1 × 10^8^ cfu ml^−1^ *Pst* DC3118 [OD_600_ = 0.1; Spectronic 20D+, Thermo Scientific] in 0.25 mM MgCl_2_ containing 0.025% Silwet-77 solution) or syringe-infiltrated with a bacterial suspension (*Pst* DC3118 [1 × 10^5^ ~ 1 × 10^6^ cfu ml^−1^] or *Pst* DC3000 (*avrRpt2*) [1 × 10^6^ cfu ml^−1^] in 0.25 mM MgCl_2_ solution). Bacterial populations were determined by serial dilutions of plant homogenates after two or three days post-inoculation. A total of 12 leaf discs (diameter of 4 mm) from three fully expanded leaves of one plant were collected as one biological replicate. For MAMP-induced protection assays, plant leaves were syringe-infiltrated with a 0.1 μM flg22 (EZBiolab), 0.5 μM elf26 (EZBiolab), or 0.1% DMSO (mock) for 22-24 hours followed by infiltration of bacterial suspension (1 × 10^6^ cfu ml^−1^ *Pst* DC3000 in 0.25 mM MgCl_2_ solution).

To assess bacterial numbers at day 0 in various mutant plants, bacterial populations were recorded 1 hour after infiltration-inoculation with 5 × 10^5^ cfu ml^−1^ *Pst* DC3118 (Fig. S1)

### Stomatal closure assays

Leaf discs (~3 mm × 3 mm) were collected one hour after lights turned on in the growth chamber and were submerged in MES buffer (25mM MES-KOH pH6.15, 10mM KCl) with 100 μM SA (Sigma-Aldrich), 10 μM ABA (Sigma-Aldrich) or 0.1% DMSO (mock) for one hour. For measuring pathogen-induced stomatal closure, leaf discs were submerged in water or bacterial suspension (1x10^8^ cfu ml^−1^ *Pst* DC3118 in water) for two hours. Stomatal apertures were captured using Olympus FluoView 1000 Spectral-based Laser Scanning Confocal Microscope with excitation/emission at 405/460 nm. The length and width of the pore aperture were measured using ImageJ (https://imagej.nih.gov/ij/). Stomata from four different plants per genotype (1-2 leaf discs per plant, ~8 stomata per leaf disc) were imaged. At least 30 stomata per genotype were measured for each treatment and the ratio of width/length was graphed to represent the stomatal aperture status.

### Phytohormone extraction and quantification

Phytohormones were extracted and quantified as described previously (Zeng et al. 2011) with minor modifications. In brief, approximately 100 mg leaf tissues from four- to five-week old plants, were flash-frozen in liquid nitrogen, grounded and extracted at 4 °C overnight using 1 ml of ice-cold extraction buffer containing methanol:water (80:20 v/v), 0.1% v/v formic acid, and 0.1 g l^−1^ butylated hydroxytoluene supplemented with 100 nM deuterated-ABA (ABA-^2^H_6_) as an internal standard. After overnight incubation at 4 °C with gentle agitation, samples were cleared by centrifugation (12000 × *g*) and filtered through a 0.2 μm PTFE membrane (Millipore) before being transferred to autosampler vials. ABA (Sigma-Aldrich) was used to prepare calibration standards and generate standard curves (3.9 nM-1000 nM). 10 uL injections of plant extracts were separated using an Acquity UPLC module (Waters) equipped with an Ascentis Express fused-core C18 Column (2.1×50 mm, 2.7 μm; Supelco) heated to 50 °C. The aqueous phase A consisted of 0.15% formic acid in water and the organic phase B was methanol. The separation consisted of a linear increase from A:B (49:1, v/v) to 100% B over 2.5 minutes at a flow rate of 0.4 ml minute^−1^. Transitions from deprotonated molecules to characteristic product ions were monitored for ABA (m/z 263.1>153.1) and ABA-^2^H_6_ (m/z 269.1>159.1) on a Quattro Premier tandem mass spectrometer (Waters) in negative ion mode. The capillary voltage was 3500 V, cone voltage 25 V, and extractor voltage 5 V. The source temperature was maintained at 120 °C and desolvation temperature was 350 °C. Nitrogen was used as desolvation and cone gas at a flow rate of 600 l h^−1^ and 50 l h^−1^, respectively. The collision energies and source cone potentials were optimized for each transition using QuanOptimize software. MassLynx 4.1 equipped with application manager QuanLynx was used for data acquisition and processing.

### Scanning electron microscopy (SEM)

SEM samples were prepared and scanned at the Center for Advanced Microscopy, MSU. Leaf samples were fixed at 4 °C for one and a half hours in 4% glutaraldehyde buffered with 0.1 M sodium phosphate at pH 7.4. Following a brief rinse in the buffer, samples were dehydrated in an ethanol series (25%, 50%, 75%, 95%) for 50-60 min each, and then in 100% ethanol for one hour three times. Samples were dried in a Leica Microsystems model EM CPD300 critical point dryer (Leica Microsystems) using liquid carbon dioxide as the transitional fluid. Samples were then mounted on aluminum stubs using high vacuum carbon tabs (SPI Supplies) and coated with osmium (~ 10 nm thickness) in an NEOC-AT osmium coater (Meiwafosis Co., Ltd.). Finally, samples were examined with a JEOL JSM-6610LV scanning electron microscope with SEI mode at 12 kV accelerating voltage, 30 spot size and ~15 mm working distance (JEOL Ltd.).

### Transmission electron microscopy (TEM)

TEM samples were prepared and scanned at the Center for Advanced Microscopy, MSU. After primary fixation (formaldehyde/glutaraldehyde, 2.5% each in 0.1 M sodium cacodylate buffer, pH 7.4, Electron Microscopy Sciences), samples were washed with 0.1 M cacodylate buffer and postfixed with 1% osmium tetroxide in 0.1 M cacodylate buffer, dehydrated in a gradient series of acetone and infiltrated and embedded in Spurr. 70-nm thin sections were obtained using a Power Tome Ultramicrotome (RMC, Boeckeler Instruments) and post-stained with uranyl acetate and lead citrate. Images were taken with JEOL 100CXII Transmission Electron Microscope (JEOL Ltd.) at an accelerating voltage of 100kV.

### Protein immunoblot analysis

Proteins were extracted from four- to five-week-old plants using protein extraction buffer (50 mM Tris-HCl, pH7.5, 150 mM NaCl, 1% NP-40, 1% sodium deoxycholate, 0.1% SDS with 100 μM MG132 proteasome inhibitor (Cayman Chemical), 1% protease inhibitor (Sigma-Aldrich) and 1 mM DTT). Protein concentrations were measured using the RC/DC protein assay kit (BioRad) with bovine serum albumin (BSA; BioRad) as standards. Protein samples were adjusted to the same concentrations of total proteins and immunoblotted with the corresponding antibodies. For evaluating the expression of MUR1-4xMyc from *35S*_*pro*_*:MUR1-4xMyc* plants, rabbit polyclonal anti-c-Myc primary antibodies (1:5,000; Clontech) and goat anti-rabbit secondary antibodies (1:20,000; Agrisera) were used. For detecting α1,3-fucosylated protein *N*-glycans, rabbit anti-fucose primary antibodies (1:4,000; Agrisera) and goat anti-rabbit secondary antibodies (1:20,000) were used. For detecting loading controls, the membranes were stained with staining solution (40% methanol, 10 % acetic acid, 0.1% (w/v) Naphthol Blue Black (Sigma-Aldrich)) for 20 min, and destained twice with destaining solution (20% methanol, 7.5% acetic acid) for 10 min each.

Immunoblot of FLS2 and BRI1-ASSOCIATED KINASE1 (BAK1) proteins, was performed as previously described (Tateda et al. 2014) with minor modifications. Briefly, 3-week old plants were harvested in liquid nitrogen and ground to fine powders before being taken up in a 3 x volume (3 ml g^−1^ tissue) of protein extraction buffer (50 mM Tris-HCl, pH 7.6, 150 mM NaCl, 10% glycerol, 1% Igepal CA-630 (Sigma-Aldrich), 0.5% sodium deoxycholate, and 1 x cOmplete mini protease inhibitor (1 tablet per 10 ml; Roche)). Crude extracts were subsequently centrifuged at 10,000 x g to pellet insoluble materials. Solubilized protein was normalized via Bradford assay (BioRad). For relative abundance comparisons, approximately 60 ug of solubilized protein was separated by SDS-PAGE on 4-12% NuPAGE bis-tris gels (ThermoFisher). For deglycosylation experiments, 20 ug of solubilized protein was treated with *N*-glycosidase F (PNGase F) (New England Biolabs) according to the manufacturer’s instructions and separated by SDS-PAGE on 3-8% NuPAGE tris-acetate gels (ThermoFisher). SDS-PAGE gels were transferred to PVDF membranes using an iBlot 2 dry blotting system (ThermoFisher) and proteins were analyzed by immunoblotting with anti-FLS2 (1:5000; Agrisera) and anti-BAK1 (1:8000; Agrisera) antibodies. Horseradish peroxidase-conjugated anti-rabbit IgG antibody (1:60000; Agrisera) was used as a secondary antibody and visualized with either SuperSignal West Dura or SuperSignal West Pico PLUS chemiluminescent substrates.

### RNA isolation and qPCR assays

Col-7 and *scord6* leaves were collected after elf26, flg22, or 0.1% DMSO (mock) treatment. Total RNA was extracted using RNeasy Plant Mini kit (Qiagen). M-MLV Reverse transcriptase (Life Technologies) and SYBR Green master mix (Life Technologies) were used for reverse transcription and real-time PCR. Primers were described in Supporting Information Table S1.

### ROS assays

Leaf discs (diameter = 4 mm) from five-week old plants were harvested and floated (the adaxial side facing up) in white 96-well microplate filled with 200 μl water per well. Water was removed 16-18 hours later. 100 μl solution (34 μg ml^−1^ luminol (Sigma-Aldrich), 20 μg ml^−1^ horseradish peroxidase (Sigma-Aldrich) in water) with 0.1 μM flg22 or 0.3% DMSO (mock) were added into each well and luminescence was measured immediately at 470 nm using SpectraMax L Luminescence Microplate Reader (Molecular Devices).

### Qualification of bacterial type III effector translocation

Bacterial effector translocation assays were performed and quantified as described previously (Huot et al. 2017). Briefly, leaves of four- to five-week old Col-7 plants were infiltration-inoculated with 5-10 × 10^7^ cfu ml^−1^ of *Pst* DC3000, which carries the P _*nptII*_ *::avrPto-CyaA* plasmid (Schechter et al. 2004). In order to achieve similar bacterial numbers between Col-7 and the *scord6* mutant after 7 hours of inoculation, leaves of *scord6* plants were infiltration-inoculated with 1-5 × 10^7^ cfu ml^−1^ of *Pst* DC3000 (P _*nptII*_ *::avrPto-CyaA*). Leaf discs were collected seven hours post inoculation for both bacterial populations and cAMP quantification. Bacterial populations were determined by serial dilutions of plant homogenates. cAMP was extracted and quantified using the Direct cAMP ELISA kit (ENZO) and normalized by total plant protein, which was quantified using a Quickstart Bradford assay (BioRad). In order to eliminate the influence of different bacterial populations, cAMP amounts (pmol cAMP μg^−1^ protein) were further divided by the corresponding bacterial populations (cfu cm^−2^) in each plant.

## Results

### Identification of the *SCORD6* gene

**Fig. 1:**
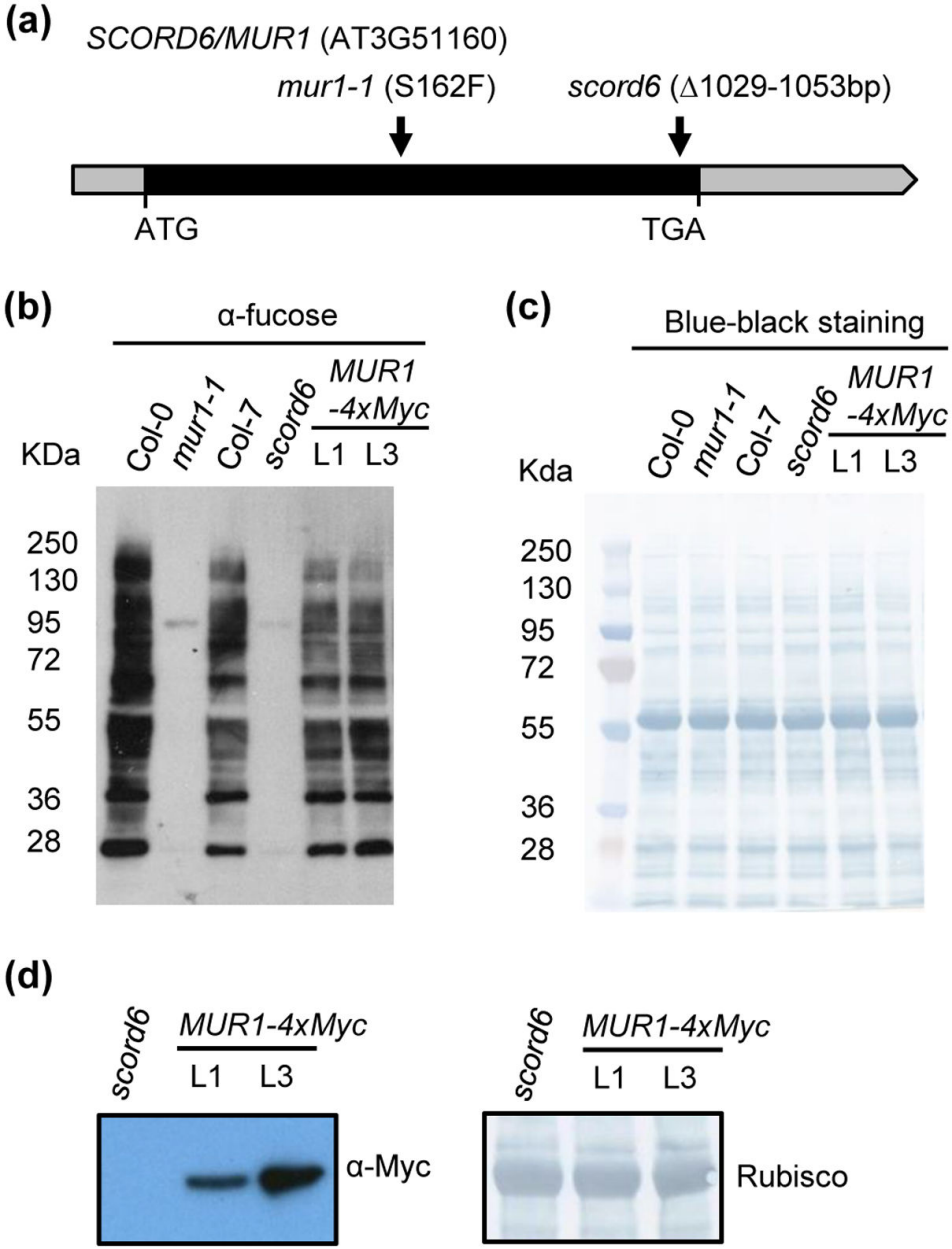
Identification of the *SCORD6* gene. (a) Schematic depiction of the Arabidopsis *SCORD6/MUR1* (AT3G51160) gene. Exon is depicted as black box, and untranslated regions (5’ UTR and 3’ UTR) are shown as gray boxes. Arrows indicate the positions of a SNP or deletion for different allelic mutant lines. (b) Western blot analysis of total leaf proteins of Col-0, *mur1-1*, Col-7, *scord6* and *35S*_*pro*_*:MUR1-4xMyc* transgenic plants using an anti-fucose antibody. (c) Naphthol Blue Black staining of the same total leaf protein samples to illustrate equal loading. (d) Western blot to detect the expression of MUR1-4xMyc protein in transgenic *35S*_*pro*_*:MUR1-4xMyc* lines in the *scord6* background.

Although the *scord6* mutant was previously isolated from the activation-tagging T-DNA (pSKI015) insertion lines (Weigel et al. 2000), the T-DNA insertion site could not be recovered using plasmid rescue or inverse genomic polymerase chain reaction (iPCR) cloning (Zeng et al. 2011). Furthermore, no T-DNA (pSKI015)-associated glufosinate/Basta resistance was detected in the *scord6* mutant (Fig. S2), indicating loss or mutation of the T-DNA sequence in this mutant. Consistent with these observations, we could not locate the intact T-DNA sequence during the analysis of *scord6* genomic sequences. We therefore hypothesized that the *scord6* mutant phenotypes may be caused by other types of sequence variants, including single-nucleotide polymorphisms (SNPs) or insertions/deletions (INDELs), in the *scord6* genome. Using SAMtools and VCFtools, homozygous variations detected only in the *scord6* genome, but not in the parental Col-7 genome, were selected and mapped back to the previously identified candidate regions by physical mapping (Zeng et al. 2011). Among the SNPs and INDELs detected, only a 25-bp deletion was located to the candidate region (18.84 Mb-19.03 Mb/III) (Zeng et al. 2011). Specifically, the deletion was found near the 3′ end of the AT3G51160 gene, previously named *MUR1* (Reiter et al. 1993) and *GMD2* (Bonin et al. 1997) (hereinafter called *MUR1* gene) (Fig. 1a). The deletion was further confirmed using IGV (Robinson et al. 2011) and genomic PCR.

### Similar _L_-fucose deficiency and stomatal phenotypes in *scord6* and *mur1-1* mutants

*MUR1* encodes an isoform of GDP-_D_-mannose-4,6-dehydratase and catalyzes the first step of the *de novo* synthesis of GDP-_L_-fucose from GDP-_D_-mannose (Bonin et al. 1997). _L_-Fucose is involved in the biosynthesis of several cell wall polymers (e.g., pectin, xyloglucan), and in the sugar-mediated modification of plant proteins (e.g., glycosylation and mono-*O*-fucosylation of DELLAs) (Reiter et al. 1993; Zentella et al. 2017). Arabidopsis *mur1* mutants (Reiter et al. 1993) exhibit an almost complete loss of _L_-fucose in shoot derived cell wall materials and glycoproteins (Reiter et al. 1993; Zablackis et al. 1996; Rayon et al. 1999; O'Neill et al. 2001; van Hengel & Roberts 2002). We used anti-fucose antibodies to detect α1,3-fucosylated protein *N*-glycans in *scord6* and *mur1-1* (with a point mutation, S162F, in the *MUR1* gene; Fig. 1a) mutants and their corresponding wild-type Col-7 and Col-0 plants. Compared to Col-7 plants, significantly less α1,3-fucosylated *N*-glycans were detected in the *scord6* leaves (Fig. 1b,c). The level of α1,3-fucosylated *N*-glycan in the *scord6* mutant was comparable to the level in the *mur1-1* mutant. We also produced stable T_2_ lines of the *scord6* mutant expressing *MUR1-4xMyc* under the control of the constitutive cauliflower mosaic virus 35S promoter *35S*_*pro*_. These lines produced the MUR1-4xMyc protein (Fig. 1d) and restored the production of α1,3-fucosylated *N*-glycans in the *scord6* mutant (Fig. 1b,c).

The *scord6* mutant was previously observed to have an abnormal stomatal morphology (Zeng et al. 2011). In this study, we found that, like the *scord6* mutant, the *mur1-1* mutant also showed markedly reduced central ridges surrounding the stomatal aperture (Fig. S3). TEM examination revealed that the collapsed central ridges of stomatal aperture are associated with an altered pattern of the outer cuticular ledges, which exhibited smaller upward angles in the *scord6* and *mur1-1* mutants compared to Col-0 plants (Fig. S3). This indicates a tight association between production of _L_-fucose and formation of stomatal cuticular ledges.

### Mutations in the *MUR1* gene affect Arabidopsis immunity

**Fig. 2:**
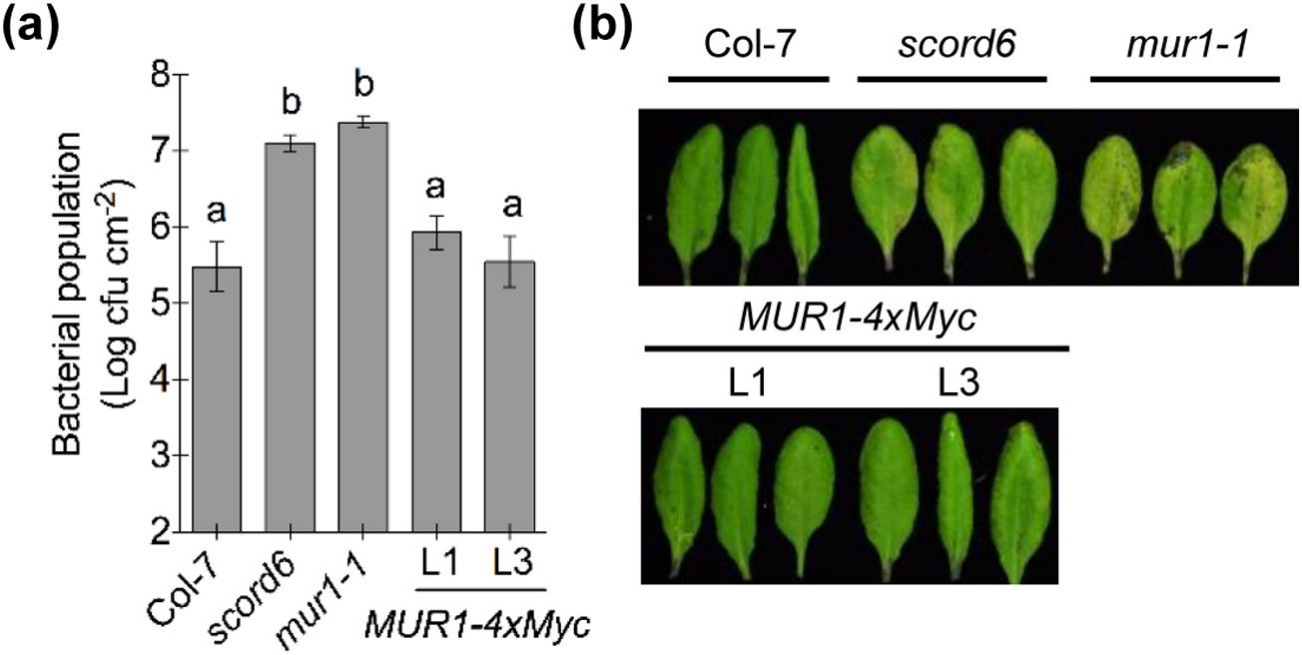
Mutations in the *MUR1* gene affect Arabidopsis defenses. (a, b) Bacterial populations (a) and disease symptoms (b) three days after dip-inoculation with 1 × 10^8^ cfu ml^−1^ *Pst* DC3118. Different letters above the columns indicate significant differences (P < 0.05) of bacterial populations between genotypes by one-way ANOVA with Tukey’s test (n = 4, error bars, ± standard error of the mean [SEM]).

The identification of *SCORD6* as *MUR1* prompted us to examine the disease susceptibility of the *mur1-1* mutant, by monitoring its stomatal response and apoplastic immune capacity. For overall disease susceptibility assays, we infected Col-7, the *scord6* mutant and the *mur1-1* mutant by dip-inoculation with the COR-deficient mutant *Pst* DC3118. Like *scord6* plants (Zeng et al. 2011), *mur1-1* plants showed increased susceptibility to *Pst* DC3118, illustrated by more severe disease symptoms and higher bacterial populations, compared to wild-type plants (Fig. 2). Importantly, the transgenic lines of *35S*_*pro*_*:MUR1-4xMyc* in the *scord6* background showed recovery of wild-type resistance against *Pst* DC3118 (Fig. 2). This genetic complementation experiment demonstrates that the 25-bp deletion mutation in the *MUR1* gene is indeed responsible for the enhanced disease susceptibility to *Pst* DC3118 in the *scord6* mutant.

Next, we examined the *mur1-1* mutant for bacteria-, SA- or ABA-induced stomatal closure. As shown in Fig. S4, wild-type Col-7 and *35S*_*pro*_*:MUR1-4xMyc* plants exhibited significantly reduced stomatal apertures in response to *Pst* DC3118 inoculation (Fig. S4a,b). In contrast, stomatal apertures of the *scord6* and *mur1-1* mutants were less closed after inoculation with *Pst* DC3118 (Fig. S4a), suggesting compromised stomatal defense. Similarly, in response to SA treatment, wild-type Col-7 showed significant stomatal closure, whereas the *scord6* and *mur1-1* mutants exhibited almost no reduction in stomatal aperture (Fig. S4c). Interestingly, wild-type Col-7, the *scord6* mutant and the *mur1-1* mutant showed normal stomatal closure in response to ABA treatment (Fig. S4d). Furthermore, the basal levels of ABA were also comparable between the *scord6* mutant and wild-type Col-7 plants (Fig. S5), in contrast to significantly lower basal levels of SA in the *scord6* mutant (Zeng et al. 2011). Thus, *mur1* mutations do not pleiotropically affect ABA-induced stomatal closure, but they specifically affect pathogen- and SA-induced stomatal closure.

To determine whether the *mur1-1* mutant also exhibited increased susceptibility in the apoplast, leaves of these mutants were syringe-infiltrated with *Pst* DC3118 to bypass stomatal defense. Both the *scord6* and *mur1-1* mutant plants showed increased bacterial growth and disease symptoms (Fig. S6). In contrast, *35S*_*pro*_*:MUR1-4xMyc* plants restricted the multiplication of *Pst* DC3118 inside the leaf apoplast to a level that was comparable to wild-type plants (Fig. S6b,c).

Taken together, the results from our genomic, molecular genetics, and phenotypic analyses are consistent with the conclusion that *SCORD6* is *MUR1*, implicating a novel role of *MUR1* in plant immunity against bacterial pathogens.

### Mutations in the *MUR1* gene affect PTI and ETI in the Arabidopsis apoplast

The increased bacterial multiplication inside the apoplast of the *scord6* mutant could be caused by a defect in a canonical defense pathway(s) or some other physiological processes, which was a significant uncertainty in our previous study of the *scord6* mutant (Zeng et al. 2011). In this study, we conducted a series of experiments to gain insight into the apoplast hyper-susceptibility of *scord6/mur1* mutants. First, we performed experiments to determine whether a lack of _L_-fucose in the cell walls of *scord6* and *mur1-1* plants affects bacterial type III secretion of effectors in the apoplast, we performed translocation assays using the AvrPto-CyaA reporter (Schechter et al. 2004). Slightly higher levels of cAMP were detected in the *scord6* mutant compared to Col-7 plants. However, this slight difference was not significant in two of the three experimental repeats (Fig. S7). Furthermore, analysis of pooled results from all three experiments did not show a significant difference between the *scord6* mutant and wild-type Col-7 plants (Fig. S7), suggesting that increased bacterial multiplication in the apoplast in the *scord6* and *mur1-1* plants is not likely caused by increased translocation of bacterial effectors.

**Fig. 3:**
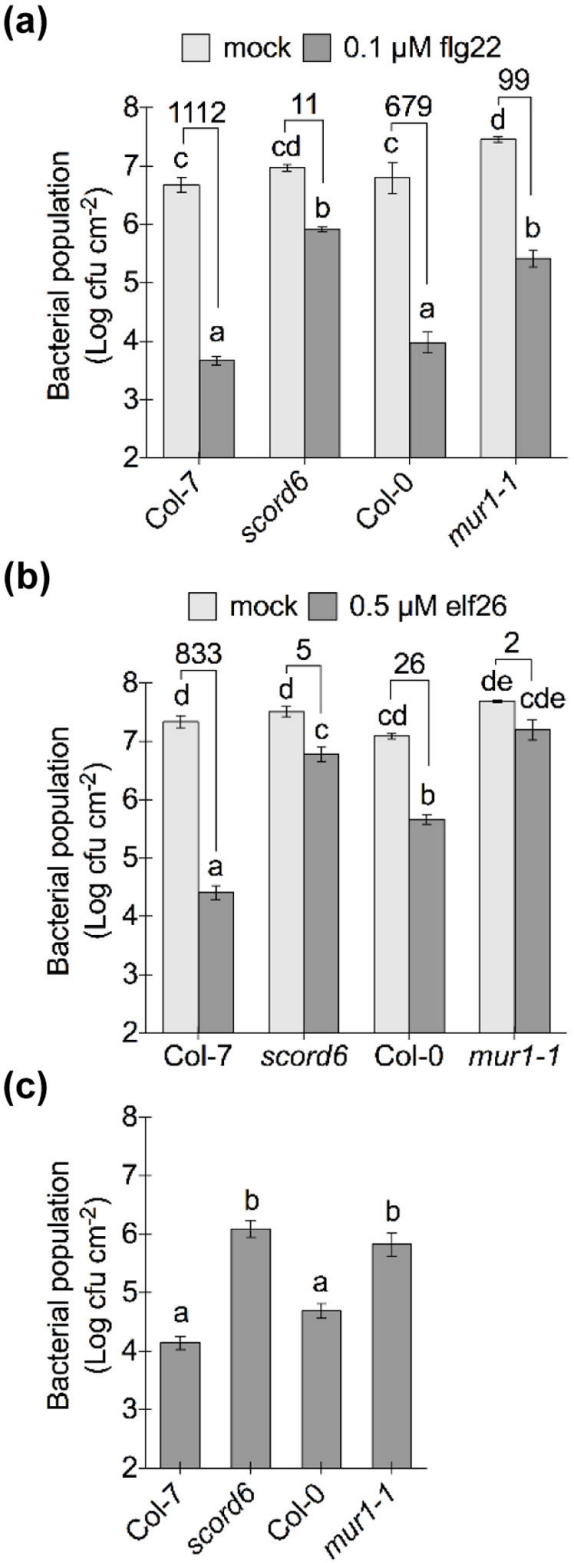
Mutations in the *MUR1* gene affect flg22-, elf26- and AvrRpt2-induced immunity in Arabidopsis. (a) Bacterial populations two days after infiltration-inoculation with 1 × 10^6^ cfu ml^−1^ *Pst* DC3000. Plants were pre-treated with 0.1 μM flg22 or 0.1% DMSO (mock) for 22 hours. Different letters above columns indicate significant differences (P < 0.05) between bacterial populations (n = 4, error bars, ± SEM); analyzed by two-way ANOVA with Tukey’s test. Numbers above the columns are fold changes of bacterial populations with flg22 treatment compared to mock treatment, indicating the inhibition of bacterial growth with flg22 treatment. (b) Bacterial populations two days after infiltration-inoculation with 1 × 10^6^ cfu ml^−1^ *Pst* DC3000. Plants were pretreated with 0.5 μM elf26 or 0.1% DMSO (mock) for 22 hours. Different letters above columns indicate significant differences (P < 0.05) between bacterial populations (n = 4, error bars, ± SEM), analyzed by two-way ANOVA with Tukey’s test. Numbers above the columns are fold changes of bacterial populations with elf26 treatment compared to mock treatment, indicating the inhibition of bacterial growth with elf26 treatment. (c) Bacterial populations two days after infiltration-inoculation with 1 x 10^6^ cfu ml^−1^ *Pst* DC3000 (*avrRpt2*). Different letters above columns indicate significant differences (P < 0.05) of bacterial population between plant genotypes (n = 4, error bars, ± SEM); analyzed by one-way ANOVA with Tukey’s test.

Next, we examined the competency of the *scord6* and *mur1-1* mutants for PTI and ETI inside the apoplast. The MAMPs flg22 and elf26 were used to induce FLS2- and EFR-mediated PTI, respectively, followed by *Pst* DC3000 infection via infiltration-inoculation directly into the apoplast. Bacterial populations in the *scord6* mutant were found to be significantly higher than those in wild-type plants with 0.1 μM flg22 or 0.5 μM elf26 pre-treatment (Fig. 3a,b), indicating compromised flg22/elf26-induced PTI in the apoplast of the *scord6* mutant. Similarly, with 0.1 μM flg22 or 0.5 μM elf26 treatment, the *mur1-1* mutant harbored significantly higher levels of bacteria compared to wild-type plants (Fig. 3a,b). In particular, bacterial populations in the *mur1-1* mutant pre-treated with 0.5 μM elf26 reached a level similar to those with mock pre-treatment, indicating an almost complete loss of elf26-induced PTI (Fig. 3b). Unlike Col-7 and the *scord6* mutant, differences in bacterial populations were noted between Col-0 and the *mur1-1* mutant with mock treatment; however, the degree of bacterial growth inhibition with flg22/elf26 treatment was much larger in wild-type Col-0 plants than that in the *mur1-1* mutant (Fig. 3a,b).

**Fig. 4:**
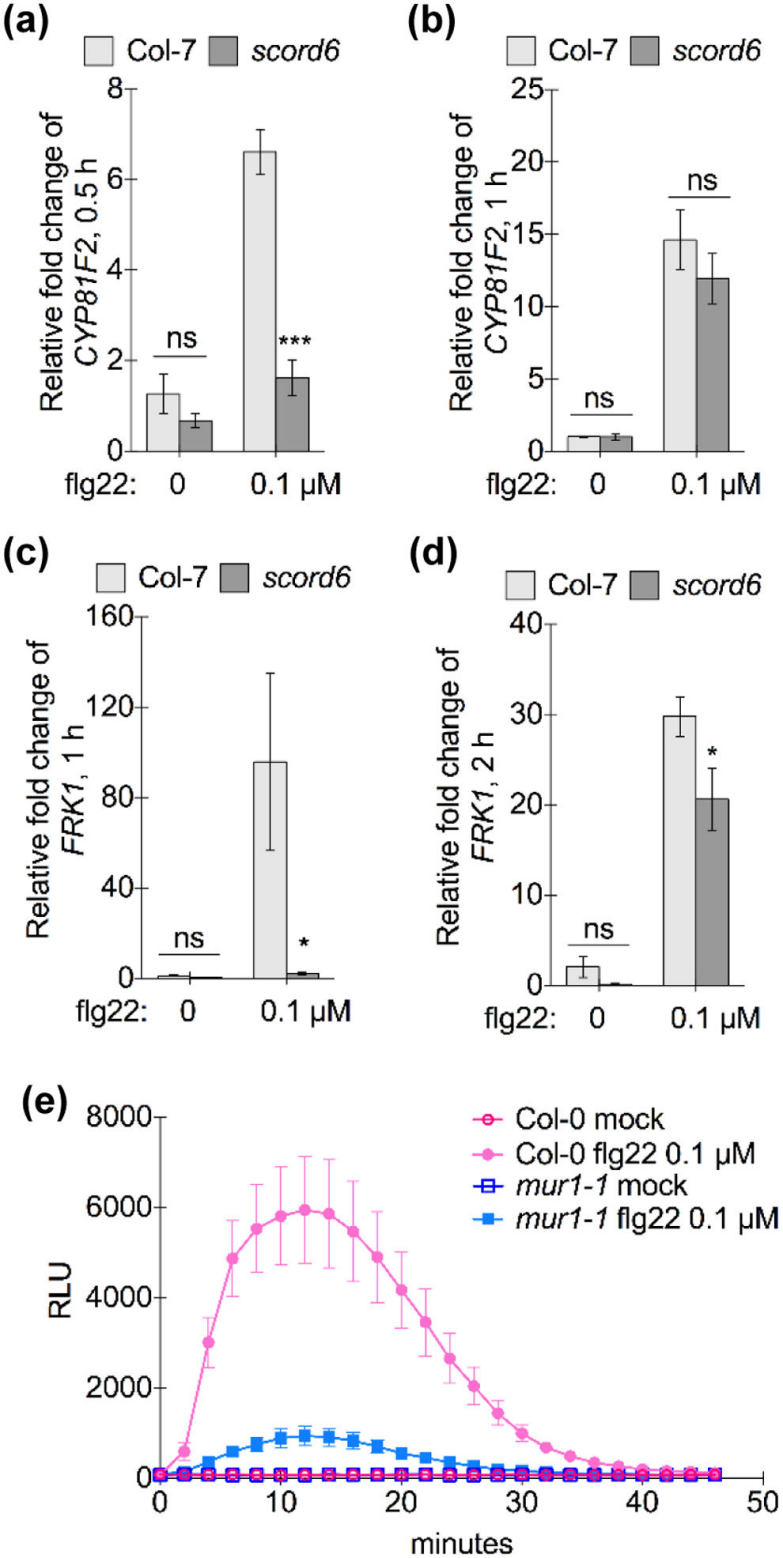
Mutations in the *MUR1* gene affect flg22-induced PTI signaling in Arabidopsis. (a, b) Expression of PTI early response gene *CYP81F2* 30 min (a) or one hour (b) after 0.1 μM flg22 or 0.1% DMSO (mock) treatment. *** P < 0.001 indicates significant differences of relative fold changes between wild-type Col-7 and the *scord6* mutant (n = 4, error bars, ± SEM), analyzed by Student's t-test (ns: not significant). (c, d) Expression of the PTI response gene, *FRK1*, one (c) or two hours (d) after 0.1 μM flg22 or 0.1% DMSO (mock) treatment. * 0.01 < P < 0.05 indicates significant differences of relative fold changes between wild-type Col-7 and the *scord6* mutant (n = 4, error bars, ± SEM), analyzed by Student's t-test (ns: not significant). (e) ROS production in Col-0 and the *mur1-1* mutant after 0.1 μM flg22 or 0.3% DMSO (mock) treatment. Relative light units (RLU) indicates the level of ROS production (n = 4, error bars, ± SEM).

To further clarify the involvement of PTI signaling components in the compromised flg22/elf26-induced protection of the *scord6* and *mur1-1* mutants, we characterized MAMP-induced expression of PTI early response genes, *CYP81F2* (AT5G57220), and *FLG22-INDUCED RECEPTOR-LIKE KINASE1* (*FRK1*, AT2G19190), in the *scord6* mutant (Asai et al. 2002; J. Li et al. 2009). 0.1 μM flg22 induced expression of the *CYP81F2* gene at 30 min in wild-type plants, while the *scord6* mutant had a significantly lower level of *CYP81F2* gene expression (Fig. 4a). One hour after flg22 treatment, however, the *scord6* mutant exhibited a similar level of *CYP81F2* expression compared to wild-type plants (Fig. 4b), indicating delayed PTI response gene expression in the *scord6* mutant. Similarly, compromised expression of the *FRK1* gene was detected in the *scord6* mutant (Fig. 4c,d). We also compared induction of ROS burst in the *mur1-1* mutant and wild-type Col-0 plants. With 0.1 μM flg22 treatment, the *mur1-1* mutant showed lower levels of ROS production compared to Col-0 (Fig. 4e). Taken together, these results indicate that the *scord6* and *mur1-1* mutants are compromised in PTI signaling.

We further investigated the abundance and the glycosylation forms of FLS2 and BAK1 in Col-7, Col-0, *scord6* and *mur1-1* plants. Compared to wild-type plants, there were no significant changes in FLS2 and BAK1 protein levels in the two mutant plants (Fig. S8). Interestingly, however, consistent with a defect in fucose biosynthesis in the *scord6* and *mur1-1* mutants, these proteins showed sensitivity to *N*-glycosidase F (PNGase F), which cleaves plant *N*-glycans without α1,3-fucose in the core *N*-acetylglucosamine (GlcNAc; Tretter et al. 1991), as indicated by a larger decrease in the molecular weights (MWs) of FLS2 and BAK1 after PNGase F. In contrast, FLS2 and BAK1 in wild-type plants were less sensitive to PNGase F treatment, as indicated by a smaller decrease in the MWs of FLS2 and BAK1. This result is consistent with the presence of α1,3-fucose in the *N*-glycans attached to FLS2 and BAK1 in wild-type plants.

To investigate whether the apoplast of the *scord6* and *mur1-1* mutants could mount an effective ETI, *Pst* DC3000 (*avrRpt2*) was infiltrated into the apoplast of the *scord6* and *mur1-1* mutants and their respective wild-type plants. Bacterial populations of *Pst* DC3000 (*avrRpt2*) were significantly higher in the *scord6* and *mur1-1* mutants, compared to their respective wild-type plants (Fig. 3c). Taken together, these results show that the apoplast of the *scord6* and *mur1-1* mutants is defective in mounting full-scale PTI or ETI against bacterial infection.

### Fucosyltransferases specific for *N*-glycans affect Arabidopsis apoplastic and stomatal defense

In plants, _L_-fucose is involved in fucosylation of a diverse set of substrates, including pectin, xyloglucan, glycoproteins, and DELLA proteins (Reiter et al. 1993; Zentella et al. 2017). Therefore, we hypothesized that the observed defect in immune responses in the *scord6* and *mur1-1* mutants could result from a defect in fucosylation of one or more of these classes of substrates. To examine this possibility, we analyzed Arabidopsis mutants that are specifically affected in each of these known fucosylation processes.

**Fig. 5:**
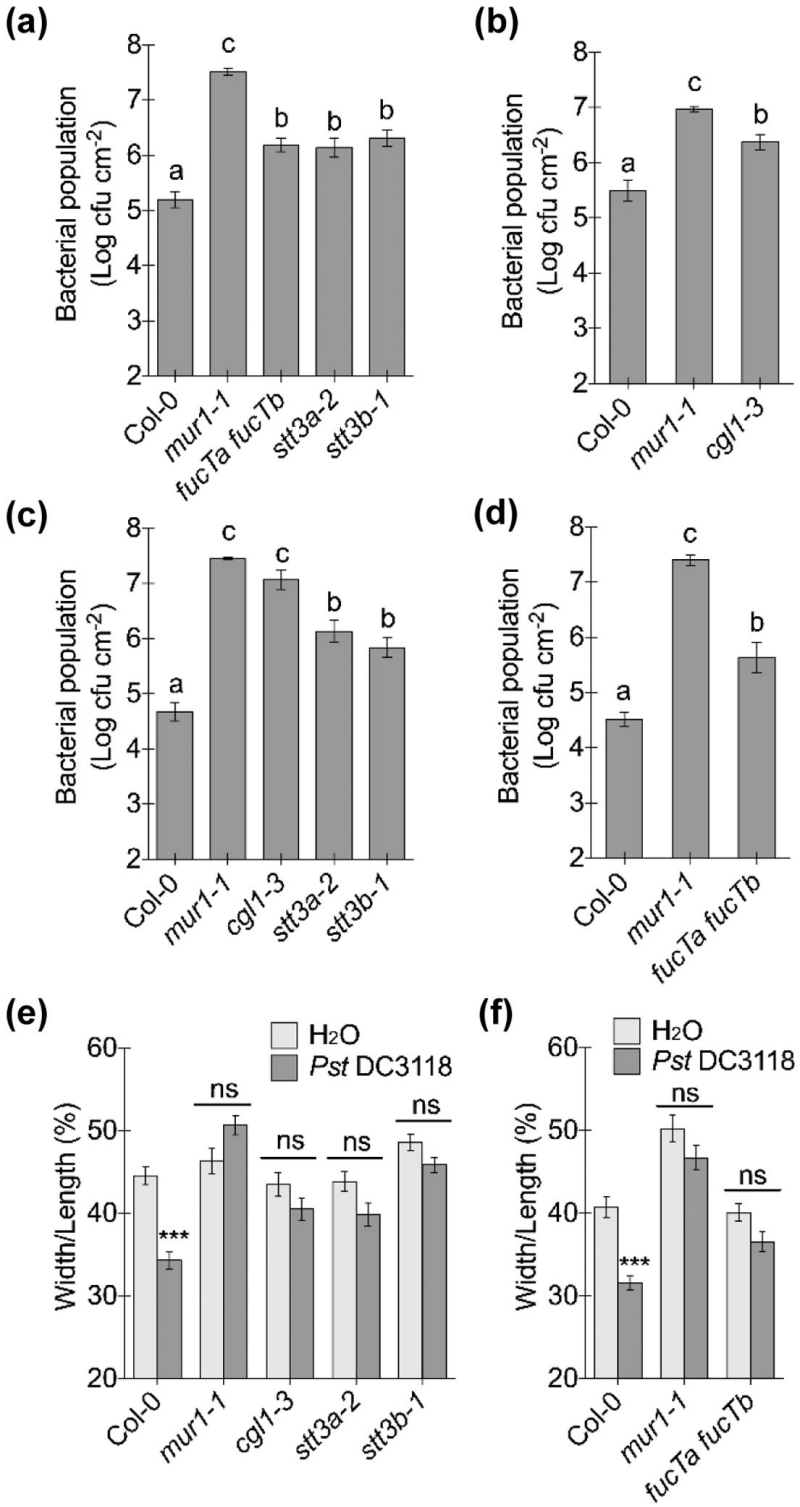
*N*-glycosylation is required for Arabidopsis stomatal closure and apoplastic defense. (a, b) Bacterial populations three days after dip-inoculation (onto the leaf surface) with 1 × 10^8^ cfu ml^−1^ *Pst* DC3118. Different letters above the columns indicate significant differences (P < 0.05) of bacterial populations between plant genotypes by one-way ANOVA with Tukey’s test (n = 4, error bars, ± SEM). (c, d) Bacterial populations three days after infiltration-inoculation (into the leaf apoplast) with 5 × 10^5^ cfu ml^−1^ *Pst* DC3118. Different letters above the columns indicate significant differences (P < 0.05) of bacterial populations between plant genotypes by one-way ANOVA with Tukey’s test (n = 4, error bars, ± SEM). (e, f) Stomatal apertures two hours after leaves were inoculated with 1 × 10^8^ cfu ml^−1^ *Pst* DC3118 or water (mock). Different letters above columns indicate significant differences (P < 0.05) between stomatal apertures (n > 50, error bars, ± SEM), analyzed by two-way ANOVA with Tukey’s test.

We first focused on fucosylation of *N*-glycan, as _L_-fucose is an important component of the carbohydrate chains of glycoproteins (Strasser 2016). Similar to the *mur1-1* mutant (Rayon et al. 1999), the *scord6* mutant is affected in the profiles of *N*-glycosylated proteins (Fig. 1b). Arabidopsis mutants defective in two core α1,3-fucosyltransferases, FucTA/FUT11 and FucTB/FUT12 (hereinafter called FucTA and FucTB), which modify *N*-glycans redundantly (Fig. S9a; Strasser et al. 2004; Strasser 2016), were examined for apoplastic and/or stomatal defenses. The *fucTa fucTb* double mutant plants showed compromised stomatal closure in response to *Pst* DC3118 inoculation (Fig. 5) and allowed increased bacterial multiplication after dip- or infiltration-inoculation, compared to wild-type plants. The increased susceptibility of the *fucTa fucTb* double mutant suggests that the core fucosyltransferases, FucTA and FucTB, are required for Arabidopsis to mount normal apoplastic and stomatal defenses. Consistent with the redundant function of FucTA and FucTB (Bakker et al. 2001), *fucTa* and *fucTb* single mutants did not exhibit enhanced susceptibility to *Pst* DC3118 infection (Fig. S10a).

Next, we examined FucTC/FUT13, which is an α1,4-fucosyltransferase involved in the synthesis of the Lewis A-glycoepitopes of *N*-glycans (Fig. S9a; Leonard et al. 2002; Strasser 2016). We found that, unlike the *fucTa fucTb* double mutant, the *fut13-2* mutant (Anderson et al. 2012) did not show increased susceptibility to *Pst* DC3118, compared to wild-type plants after dip-inoculation with *Pst* DC3118 (Fig. S10a), indicating that, unlike the core α1,3-linked fucose, the integrity of the Lewis A-type structure of *N*-glycans is not required for Arabidopsis defenses.

The requirement of FucTA and FucTB for Arabidopsis defenses prompted us to further examine the involvement of other steps of *N*-glycan processing (Fig. S9a) in plant immunity. Arabidopsis *stt3a-2* and *stt3b-1* mutants are defective in the putative subunits of the oligosaccharyltransferase (OST) complex, which catalyzes the transfer of the pre-assembled oligosaccharide to an asparagine residue (Koiwa et al. 2003). These two mutants showed increased susceptibility to *Pst* DC3118 both in dip- and infiltration-inoculation experiments. Moreover, compared to wild-type plants, stomatal responses to *Pst* DC3118 were also compromised in the *stt3a-2* and *stt3b-1* mutants (Fig. 5). Additionally, β1,2-*N*-acetylglucosaminyltransferase I (GnTI) initiates the formation of complex and hybrid *N*-glycans in the Golgi (von Schaewen et al. 1993). An Arabidopsis *GnTI* mutant, *complex glycan1* (*cgl1-3*) (Frank et al. 2008), also exhibited compromised apoplastic and stomatal defenses (Fig. 5). These results clearly showed that multiple steps of *N*-glycan processing affect apoplastic and stomatal defenses in Arabidopsis.

### Fucosyltransferases specific for *O*-glycans and xyloglucan exhibit differential effects on Arabidopsis immunity

**Fig. 6:**
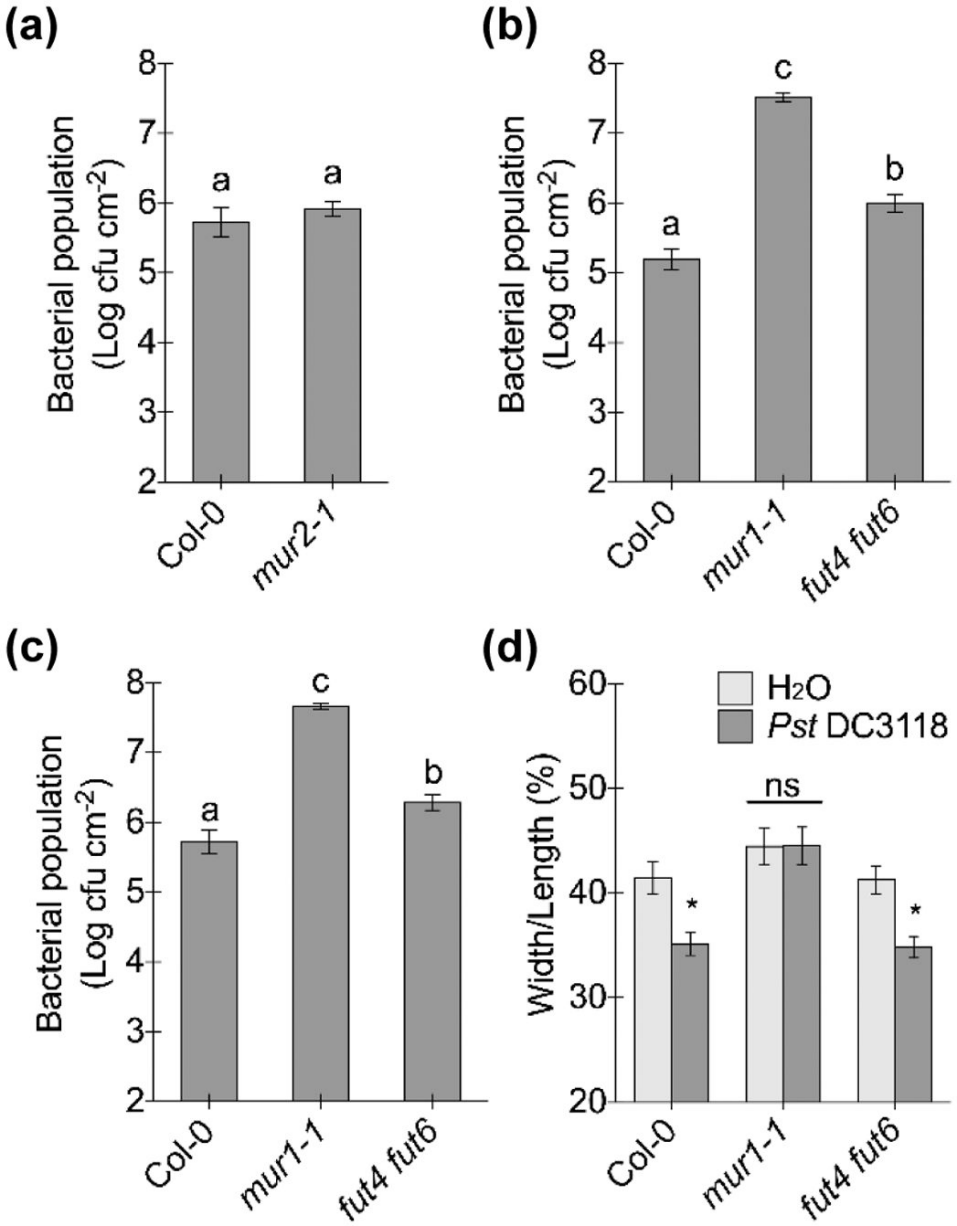
Arabidopsis fucosyltransferase mutants differentially affect apoplastic and/or stomatal defenses. (a, b) Bacterial populations three days after dip-inoculation with 1 × 10^8^ cfu ml^−1^ *Pst* DC3118. Different letters above the columns indicate significant differences (P < 0.05) of bacterial populations between plant genotypes by Student's t-test (a) or one-way ANOVA with Tukey’s test (b) (n = 4, error bars, ± SEM). Note: data from Fig. 5a and 6b were collected in the same experiment. (c) Bacterial populations three days after infiltration-inoculation with 5 × 10^5^ cfu ml^−1^ *Pst* DC3118. Different letters above the columns indicate significant differences (P < 0.05) of bacterial populations between plant genotypes by one-way ANOVA with Tukey’s test (n = 8, error bars, ± SEM). (d) Stomatal apertures two hours after leaves were inoculated with 1 × 10^8^ cfu ml^−1^ *Pst* DC3118 or water (mock). Different letters above columns indicate significant differences (P < 0.05) between stomatal apertures (n > 50, error bars, ± SEM), analyzed by two-way ANOVA with Tukey’s test.

We next carried out experiments to determine whether defects in fucosylation of cell wall polymers or *O*-glycan play a role in Arabidopsis immunity. As no pectin-specific fucosyltransferase has been found so far, disease assays and stomatal assays were conducted with Arabidopsis mutants *mur2-1* (defective in fucosylation of xyloglucan; Perrin et al. 1999; Faik et al. 2000; Vanzin et al. 2002) and *fut4 fut6* (defective in fucosylation of *O*-glycan chains of arabinogalactan proteins; Wu et al. 2010; Liang et al. 2013) (Fig. S9b,c). *mur2-1*, *fut4* and *fut6* single mutants allowed similar levels of bacterial growth as wild-type plants after *Pst* DC3118 dip-inoculation (Fig. 6a; S10b). However, the *fut4 fut6* double mutant exhibited increased bacterial growth compared to wild-type plants after both dip- and infiltration-inoculations while maintaining a normal stomatal closure response upon *Pst* DC3118 inoculation (Fig. 6b-d). These results indicate that a defect in fucosylation of xyloglucan (in the *mur2-1* mutant) does not affect Arabidopsis defenses, whereas a defect in fucosylation of *O*-glycan (in the *fut4 fut6* mutant) affects apoplastic but not stomatal defense.

### The *O*-fucosyltransferase SPY affects Arabidopsis immunity

**Fig. 7:**
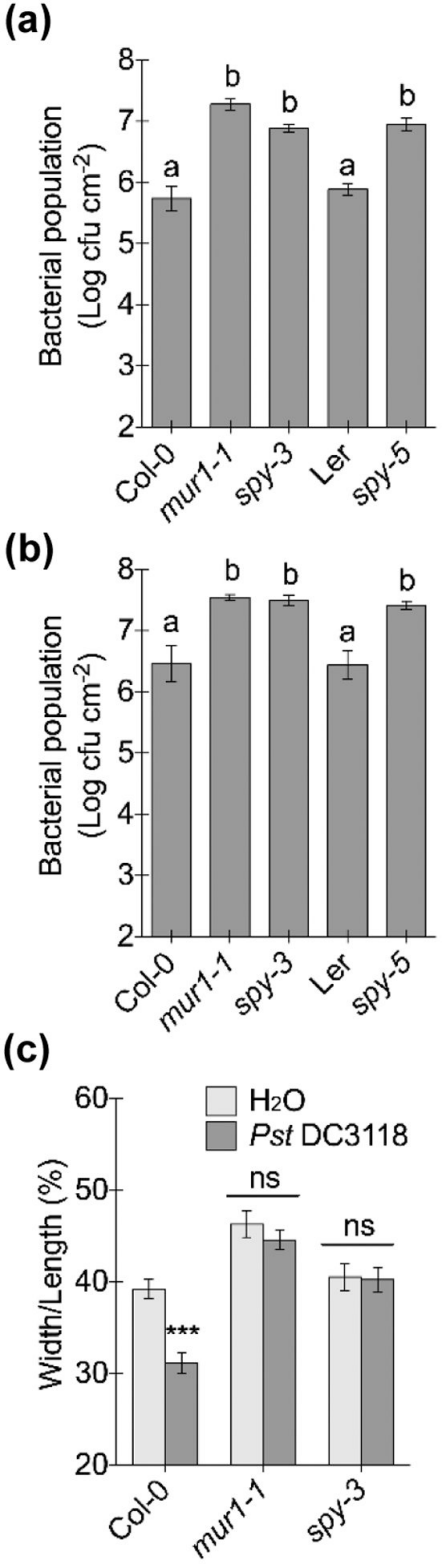
The Arabidopsis mono-*O*-glucosyltransferase *spy* mutant is affected in apoplastic and stomatal defenses. (a) Bacterial populations three days after dip-inoculation with 1 × 10^8^ cfu ml^−1^ *Pst* DC3118. Different letters above the columns indicate significant differences (P < 0.05) of bacterial populations between plant genotypes by one-way ANOVA with Tukey’s test (n = 4, error bars, ± SEM). (b) Bacterial populations three days after infiltration-inoculation with 1 × 10^6^ cfu ml^−1^ *Pst* DC3118. Different letters above the columns indicate significant differences (P < 0.05) of bacterial populations between plant genotypes by one-way ANOVA with Tukey’s test (n = 4, error bars, ± SEM). (c) Stomatal apertures two hours after leaves were inoculated with 1 × 10^8^ cfu ml^−1^ *Pst* DC3118 or water (mock). Different letters above columns indicate significant differences (P < 0.05) between stomatal apertures (n > 50, error bars, ± SEM), analyzed by two-way ANOVA with Tukey’s test.

Recently, a protein *O*-fucosyltransferase, SPINDLY (SPY), was shown to catalyze mono-*O*-fucosylation (attaching mono fucose to specific Ser/Thr residues) of DELLA transcriptional repressors (Zentella et al. 2017). This fucosylation activates DELLA repressors, which negatively regulate gibberellin (GA) hormone signaling (Zentella et al. 2017). Interestingly, DELLA proteins have been shown to be involved in plant immunity. For example, a quadruple loss-of-function mutant of DELLAs showed higher resistance to *Pst* DC3000 compared to wild-type plants (Navarro et al. 2008). Hence, we examined the immune competence of Arabidopsis *SPY* mutants, *spy-3* and *spy-5* (Jacobsen & Olszewski 1993; Wilson & Somerville 1995; Jacobsen et al. 1996). Based on pathogen inoculation (both dip and apoplastic infiltration) and stomatal closure assays, *spy* mutants exhibited compromised apoplastic and stomatal defenses (Fig. 7), compared to the corresponding wild-type plants, Col-0 and Landsberg erecta (Ler), respectively. Strikingly, *Pst* DC3118 populations in *spy-3* and *spy-5* mutants after dip- or infiltration-inoculation reached levels comparable to those in the *mur1-1* mutants. This phenotype contrasts with those of other fucosyltransferase mutants (e.g., *fucTa fucTb* and *fut4 fut6*), which exhibited only moderately enhanced disease susceptibility. This finding suggests that lack of SPY-mediated mono-*O*-fucosylation of proteins might be a significant contributor to compromised Arabidopsis immunity in the *scord6* and *mur1-1* mutants.

## Discussion

In this study, we have identified the *SCORD6* gene and provided multiple lines of evidence that *SCORD6* is in fact *MUR1*. Prior to this study, MUR1 has been known as an isoform of GDP-_D_-mannose-4,6-dehydratase catalyzing the *de novo* biosynthesis of _L_-fucose and being involved in fucosylation of xyloglucan, pectin, glycoproteins and DELLA proteins (Reiter et al. 1993; Bonin et al. 1997; Zentella et al. 2017). In this study we found that the *scord6/mur1* mutants are affected in multiple aspects/pathways of plant defense, including stomatal defense, apoplastic defense, PTI and ETI, suggesting a multifaceted role of _L_-fucose in plant immunity (Table S2).

Because Arabidopsis *mur1* mutants were originally isolated in a study of plant cell walls (Reiter et al. 1993), most studies of MUR1 have since focused on its function in cell wall composition, integrity, mechanical performance and cell-wall related plant growth and development (Reiter et al. 1993; Zablackis et al. 1996; Reiter et al. 1997; Rayon et al. 1999; O'Neill et al. 2001; van Hengel & Roberts 2002; Freshour et al. 2003; Ryden et al. 2003; Abasolo et al. 2009; Dumont et al. 2015; Goncalves et al. 2017; Voxeur et al. 2017). However, two studies have implicated a role of MUR1 in plant responses to biotic or abiotic stresses. The *mur1-1* mutant was found to exhibit significantly decreased resistance to the penetration of the non-host barley powdery mildew fungus *Blumeria graminis f. sp. hordei* (*Bgh*), which was attributed to a defect in the physical barrier of the primary cell wall (Assaad et al. 2004). Similarly, Feng and colleagues (Feng et al. 2018) found that *mur1* mutants showed hypersensitivity to salinity stress, which was again proposed to result from a defect in pectin cross-linking.

It is plausible that the requirement of *MUR1* for bacterium-triggered stomatal movements resides in part through its role in building a proper plant cell wall. Indeed, it is generally believed that guard cell walls need to be both strong and elastic in order to sustain the high internal pressure and reversible movements (Jones et al. 2005). Cell wall components, like cellulose, hemicellulose and pectin, have all been shown to be involved in basic guard cell movement in response to abiotic stress or chemical treatments (Jones et al. 2003; Jones et al. 2005; Choi et al. 2011; Merced & Renzaglia 2014; Amsbury et al. 2016; Rui & Anderson 2016). In our previous study, we reported that the *scord6* mutant has greatly reduced outer cuticular ledges of guard cells (Zeng et al. 2011). This striking stomatal phenotype is also present in the *mur1-1* mutant, further implicating an important role of *MUR1* in the formation of normal outer cuticular ledges of guard cells (Fig. S3). The outer cuticular ledges of guard cells have long been reported to function by preventing water loss (Schonherr & Ziegler 1975; Lu et al. 2012). Moreover, Pautov and colleagues recently proposed that the outer ledges prevent wide opening of the stomatal pore in woody plants (Pautov et al. 2017). Further studies involving new cuticular ledge mutants that do not affect other cellular processes could be used to determine whether an intact cuticular ledge is necessary for stomatal defense against pathogen invasion. It is important to note that *mur1* and *scord6* mutations do not pleiotropically affect stomatal movements, as ABA-triggered stomatal closure remains normal (Fig. S4). Therefore, a nonspecific effect of cell wall defects on stomatal movements would not explain the specific effect of *mur1* and *scord6* mutations on bacterium-triggered stomatal closure.

Indeed, our results suggested that MUR1 likely also functions in mediating plant immunity via other mechanisms that are indicative of immune and hormone signaling pathways (i.e., besides the requirement of MUR1 for plant immunity through the function of physical barriers or guard cell responses). We observed delayed/reduced expression of PTI early response genes and compromised ROS burst in the *scord6* or *mur1-1* mutant, respectively (Fig. 4). We found this result intriguing as previous studies showed that the EFR receptor is highly glycosylated and this glycosylation is necessary for the stability and function of EFR (J. Li et al. 2009; Nekrasov et al. 2009; Saijo et al. 2009; Haweker et al. 2010; Sun et al. 2012). An *N*-glycan processing mutant, *stt3a-2*, harbors reduced levels of EFR protein and exhibited compromised defense against *Pst* DC3000 with spray-inoculation (Saijo et al. 2009; Haweker et al. 2010). In this study, we found that *stt3a-2* and other *N*-glycan processing mutants (*stt3b-1*, *cgl1-3* and *fucTa fucTb*) showed compromised defense against *Pst* DC3118 in both dip- and infiltration-inoculations (Fig. 5) and that both FLS2 and BAK1 in the *scord6* and *mur1-1* mutants were sensitive to PNGase F, which cleaves plant *N*-glycans without α1,3-fucose in the core GlcNAc (Fig. S8). However, the involvement of fucosylation in PTI likely goes beyond affecting receptor glycosylation. This is because the triple mutant *fucTa fucTb xylT*, defective in α1,3-fucosyltransferases and β1,2-xylosyltransferase, was reported to have a wild-type level of EFR protein (Haweker et al. 2010), but we observed compromised resistance against *Pst* DC3118 in the *fucTa fucTb* double mutant (Fig. 5a,d).

It is worth noting that possible structural changes in cell wall, due to lack of fucose, might have an impact on plant immune signaling. For example, FERONIA (FER), the malectin-like receptor kinase, maintains cell-wall integrity during salt stress and appears to act as a scaffold to modulate FLS2-BAK1 receptor kinase complex assembly and plant immunity (Stegmann et al. 2017; Feng et al. 2018). Moreover, internalization of flg22-activated FLS2 depends on cytoskeleton (Robatzek et al. 2006) and is required for long distance transport of flg22 (Jelenska et al. 2017). Chemicals that perturb cell wall deposition or cell-wall related mutants have been reported to affect cytoskeleton organization (Baluska et al. 2003; Tolmie et al. 2017). Hence, it is possible that cell wall integrity may play a role in plant immunity through regulation of cell wall-associated receptors and the cytoskeleton.

Another interesting observation made in this study is the significantly compromised plant immunity in *spy* mutants. In particular, the immune defect in the *spy* mutant against *Pst* DC3118 infection was severe and comparable to that observed for the *mur1-1* mutant (Fig. 7). As other fucosyltransferase mutants (e.g., *fucTa fucTb* and *fut4 fut6*) tested in our study only exhibited moderately enhanced bacterial growth, it is likely that SPY-mediated mono-*O*-fucosylation of proteins plays a critical role in mediating plant immunity. Although SPY-mediated mono-*O*-fucosylation activates DELLAs and in turn suppresses GA signaling (Zentella et al. 2017), the increased susceptibility to *Pst* DC3118 of *spy* mutants probably result from a defect in a pathway other than GA signaling, because a quintuple mutant of DELLA proteins, *della* (Feng et al. 2008), showed resistance to *Pst* DC3118, similar to wild-type Ler plants (Fig. S10c). Moreover, higher resistance to *Pst* DC3000 was detected in the quadruple DELLA (Navarro et al. 2008), further suggesting that loss of DELLAs at least does not affect Arabidopsis immunity against *P. syringae*. These findings therefore raise the possibility that the SPY protein mediates plant immunity through pathways other than GA signaling, and besides DELLAs, there are likely other substrates of the SPY protein.

In summary, identification of *SCORD6* as *MUR1* led us to uncover a novel and multifaceted role of _L_-fucose biosynthesis and protein fucosylation genes in plant immunity against bacterial pathogen *P. syringae* and connect _L_-fucose to central plant immunity pathways, including PTI and ETI. These results open new avenues for future studies to identify immune-related targets of fucosylation and has potential to reveal new insights into immune-related polysaccharides or proteins in plants.

## Supplementary Material

Supplementary Material Fig. S1: Bacterial populations 1 hour after infiltration-inoculation with *Pst* DC3118.
Fig. S2: Loss of Basta resistance in the *scord6* mutant.
Fig. S3: SEM and TEM images of stomatal apertures.
Fig. S4: Mutations in the *MUR1* gene affect pathogen- and SA-induced stomatal closure in Arabidopsis.
Fig. S5: ABA levels in Col-7 and the *scord6* mutant.
Fig. S6: Mutations in the *MUR1* gene affect Arabidopsis apoplastic defense.
Fig. S7: Bacterial effector translocation in Col-7 and the *scord6* mutant plants.
Fig. S8: FLS2 and BAK1 abundance and sensitivity to PNGase F.
Fig. S9: Simplified diagrams of *N*-glycan processing and modification of *O*-glycan and xyloglucan.
Fig. S10: Disease assays of Arabidopsis mutants of fucosyltransferases and quintuple *della* mutant.
Table S1: List of primers.
Table S2: Summary of key assay results of the Arabidopsis mutants analyzed in this study.
