# Supplementary material for: An important role of L-fucose biosynthesis and protein fucosylation genes in Arabidopsis immunity
Source: New Phytol. Author manuscript; Available in PMC 2023 Jul 19. (PMC10354811; doi:10.1111/nph.15639)
Supplement: Supplementary Material — Fig. S1: Bacterial populations 1 hour after infiltration-inoculation with Pst DC3118. Fig. S2: Loss of Basta resistance in the scord6 mutant. Fig. S3: SEM and TEM images of stomatal apertures. Fig. S4: Mutations in the MUR1 gene affect pathogen- and SA-induced stomatal closure in Arabidopsis. Fig. S5: ABA levels in Col-7 and the scord6 mutant. Fig. S6: Mutations in the MUR1 gene affect Arabidopsis apoplastic defense. Fig. S7: Bacterial effector translocation in Col-7 and the scord6 mutant plants. Fig. S8: FLS2 and BAK1 abundance and sensitivity to PNGase F. Fig. S9: Simplified diagrams of N-glycan processing and modification of O-glycan and xyloglucan. Fig. S10: Disease assays of Arabidopsis mutants of fucosyltransferases and quintuple della mutant. Table S1: List of primers. Table S2: Summary of key assay results of the Arabidopsis mutants analyzed in this study. [file NIHMS1914007-supplement-Supplementary_Material.pdf]

***New Phytologist* Supporting Information**

Article title: An important role of L-fucose biosynthesis and protein fucosylation genes in Arabidopsis immunity

Authors: Li Zhang, Bradley C. Paasch, Jin Chen, Brad Day and Sheng Yang He

Article acceptance date: 1 December 2018

The following Supporting Information is available for this article:

Fig. S1: Bacterial populations 1 hour after infiltration-inoculation with *Pst* DC3118.

Fig. S2: Loss of Basta resistance in the *scord6* mutant.

Fig. S3: SEM and TEM images of stomatal apertures.

Fig. S4: Mutations in the *MUR1* gene affect pathogen- and SA-induced stomatal closure in Arabidopsis.

Fig. S5: ABA levels in Col-7 and the *scord6* mutant.

Fig. S6: Mutations in the *MUR1* gene affect Arabidopsis apoplastic defense.

Fig. S7: Bacterial effector translocation in Col-7 and the *scord6* mutant plants.

Fig. S8: FLS2 and BAK1 abundance and sensitivity to PNGase F.

Fig. S9: Simplified diagrams of *N*-glycan processing and modification of *O*-glycan and xyloglucan.

Fig. S10: Disease assays of Arabidopsis mutants of fucosyltransferases and quintuple *della* mutant.

Table S1: List of primers.

Table S2: Summary of key assay results of the Arabidopsis mutants analyzed in this study.

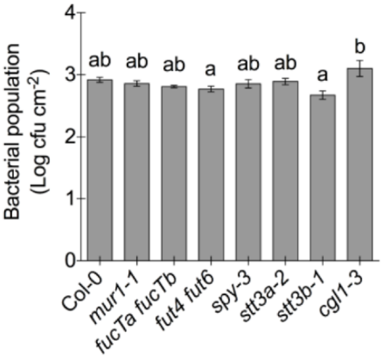

Fig. S1: Bacterial populations 1 hour after infiltration-inoculation with *Pst* DC3118.

Bacterial populations in Arabidopsis mutants were examined 1 hour after infiltration-inoculation with 5×10^5 cfu ml^{-1} *Pst* DC3118. Different letters above the columns indicate significant differences ($P < 0.05$) of bacterial populations, analyzed by one-way ANOVA with Tukey's test ($n = 4$, error bars, ± SEM).

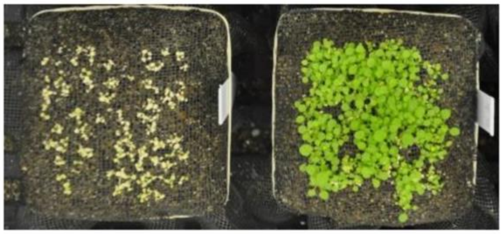

Fig. S2: Loss of Basta resistance in the *scord6* mutant.

Death of the Arabidopsis *scord6* mutant plants two weeks after sprayed with Basta solution (0.12 g l^{-1}, 0.025% Silwet-77). The *scord7* mutant (Zeng *et al.* 2011) was used as a Basta-resistant control.

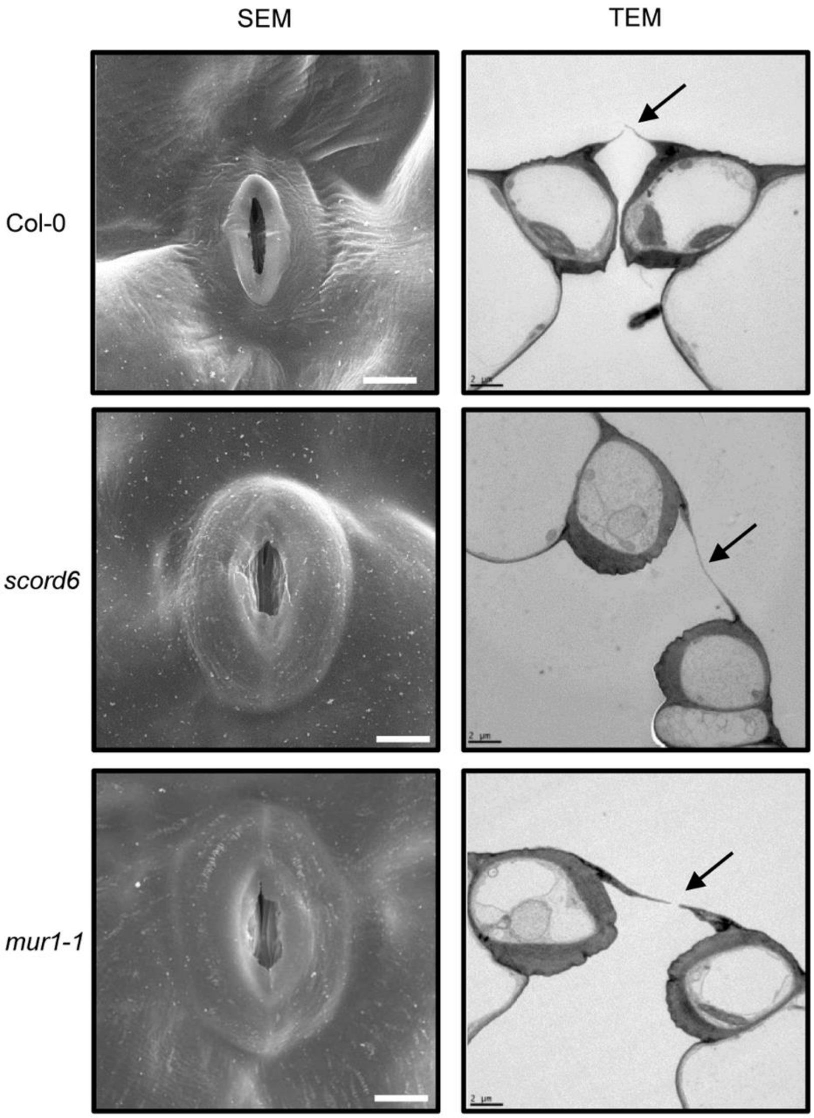

Fig. S3: SEM and TEM images of stomatal apertures.

Left, SEM images of Arabidopsis stomata, scale bar = 5 μm. Right, TEM images of the cross sections of Arabidopsis stomatal guard cells, scale bar = 2 μm. Arrows indicate the merging point of the outer cuticular ledges of two guard cells.

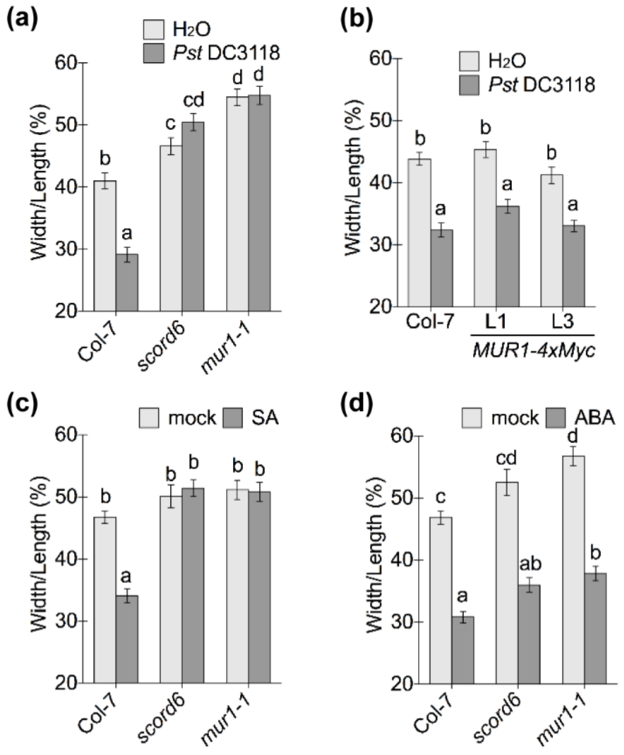

Fig. S4: Mutations in the *MUR1* gene affect pathogen- and SA-induced stomatal closure in Arabidopsis.

(a, b) Stomatal apertures two hours after leaves were inoculated with 1×10^8 cfu ml^{-1} *Pst* DC3118 or water (mock). Different letters above columns indicate significant differences ($P < 0.05$) between stomatal apertures ($n > 30$, error bars, ± SEM), analyzed by two-way ANOVA with Tukey's test.

(c, d) Stomatal apertures one hour after leaves have been exposed to 100 µM SA (c), 10 µM ABA (d) or MES buffer (mock) treatments. Different letters above columns indicate significant differences ($P < 0.05$) between stomatal apertures ($n > 30$, error bars, ± SEM), analyzed by two-way ANOVA with Tukey's test.

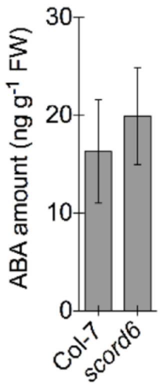

Fig. S5: ABA levels in Col-7 and the *scord6* mutant.

Hormone levels of ABA in Arabidopsis plants Col-7 and the *scord6* mutant. No significant differences were detected between the hormone levels of Col-7 and the *scord6* mutant, via Student's t-test (n = 6, error bars, ± SEM).

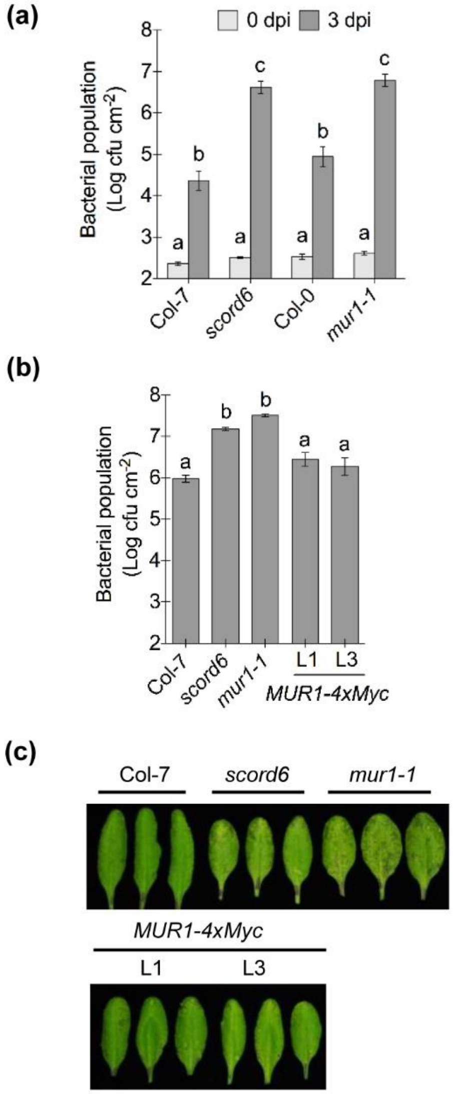

Fig. S6: Mutations in the *MUR1* gene affect Arabidopsis apoplastic defense.

(a) Bacterial populations 1 hour (Day 0) or 3 days (Day 3) after infiltration-inoculation (into the leaf apoplast) with 1×10^5 cfu ml^{-1} *Pst* DC3118. Different letters above the columns indicate significant differences ($P < 0.05$) of bacterial populations, analyzed by two-way ANOVA with Tukey's test ($n = 4$, error bars, ± SEM).

(b, c) Bacterial populations (b) and disease symptoms (c) three days after infiltration-inoculation (into the leaf apoplast) with 1×10^5 cfu ml^{-1} *Pst* DC3118. Different letters above the columns indicate significant differences ($P < 0.05$) of bacterial populations between plant genotypes by one-way ANOVA with Tukey's test ($n = 4$, error bars, ± SEM).

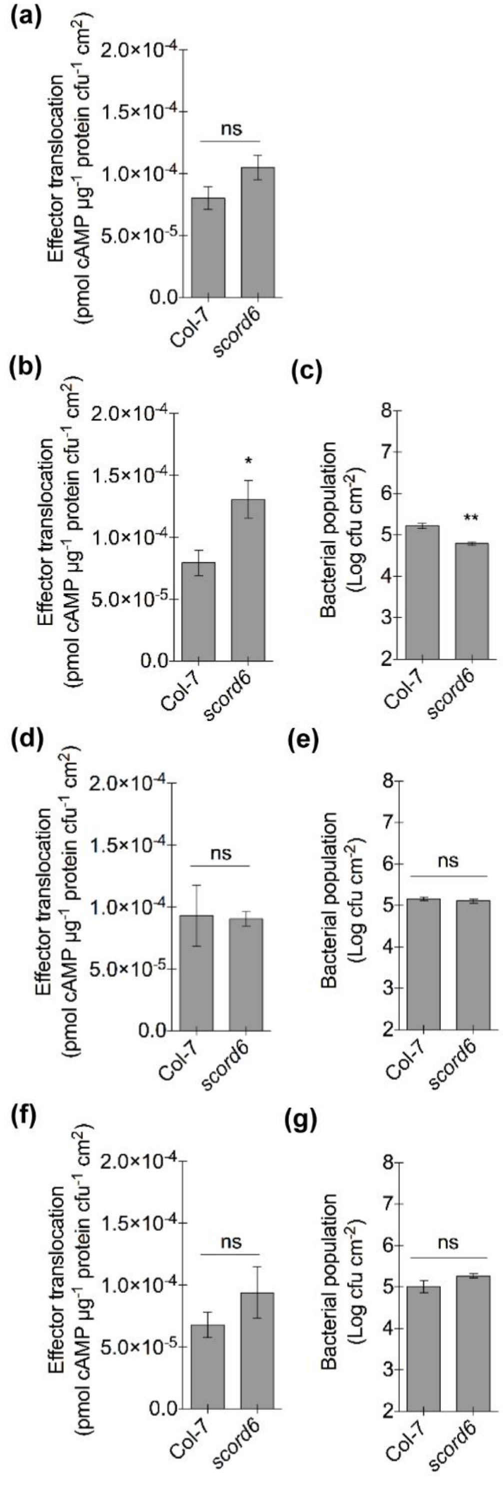

Fig. S7: Bacterial effector translocation in Col-7 and the *scord6* mutant plants.

(a) Pooled bacterial effector translocation data from three experimental repeats with total n = 12 (4 biological replicates [Arabidopsis plants] per experiment). Error bars, ± SEM. No significant

differences were detected, as analyzed by Student's t-test. The bacterial effector translocation was examined seven hours post inoculation with *Pst* DC3000 carrying the P_{nptII} *::avrPto-CyaA* plasmid. Translocation of effector protein AvrPto was represented by the cAMP amount (pmol cAMP μg^{-1} protein) divided by the corresponding bacterial number (cfu cm^{-2}) in each sample.

(b-g) Three different experimental repeats of the bacterial effector translocation in Col-7 and the *scord6* mutant (b, d, f), and the corresponding bacterial populations seven hours after infiltration-inoculation (c, e, g) (n = 4; error bars, ± SEM). *0.01 < P < 0.05 indicates a significant difference of cAMP amounts between wild-type Col-7 and the *scord6* mutant, as analyzed by Student's t-test; ns: not significant. **0.001 < P < 0.01 indicates a significant difference of bacterial populations between wild-type Col-7 and the *scord6* mutant, as analyzed by Student's t-test; ns: not significant.

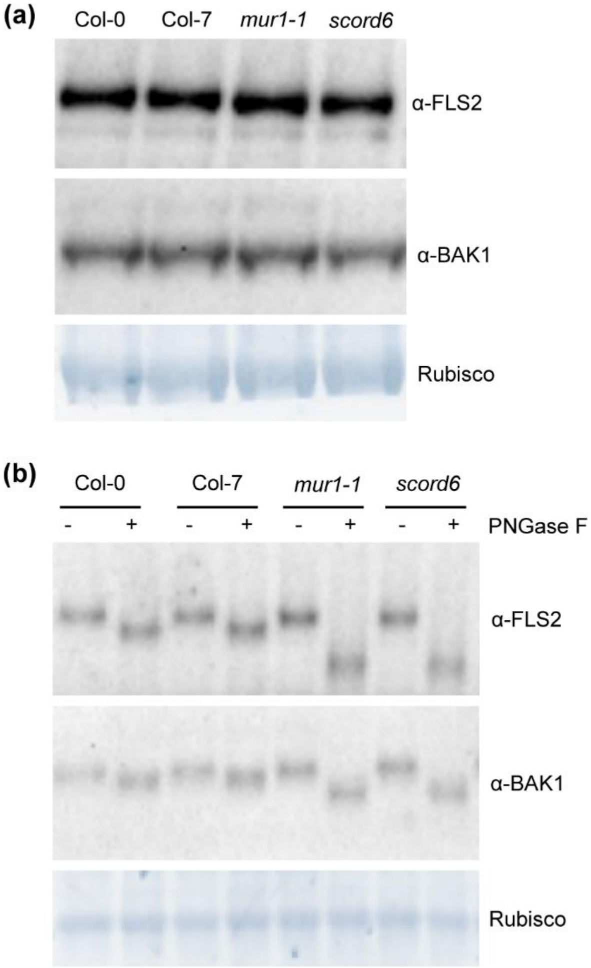

Fig. S8: FLS2 and BAK1 abundance and sensitivity to PNGase F.

(a) The first two panels: Western blot of total Arabidopsis leaf proteins for FLS2 and BAK1 in wild-type, *scord6* and *mur1-1* mutant plants using α-FLS2 and α-BAK1 antibodies. Lower panel: Naphthol Blue Black staining of gel showing the Rubisco large subunit as a loading control.

(b) The first two panels: Western blot of total Arabidopsis leaf proteins for FLS2 and BAK1 after PNGase F. Lower panel: Naphthol Blue Black staining of gel showing the Rubisco large subunit as a loading control. Please note that FLS2 and BAK1 proteins showed a larger decrease in the MWs of FLS2 and BAK1 after PNGase F. In contrast, FLS2 and BAK1 in wild-type plants were less sensitive to PNGase F treatment, as indicated by a smaller decrease in the MWs of FLS2 and BAK1.

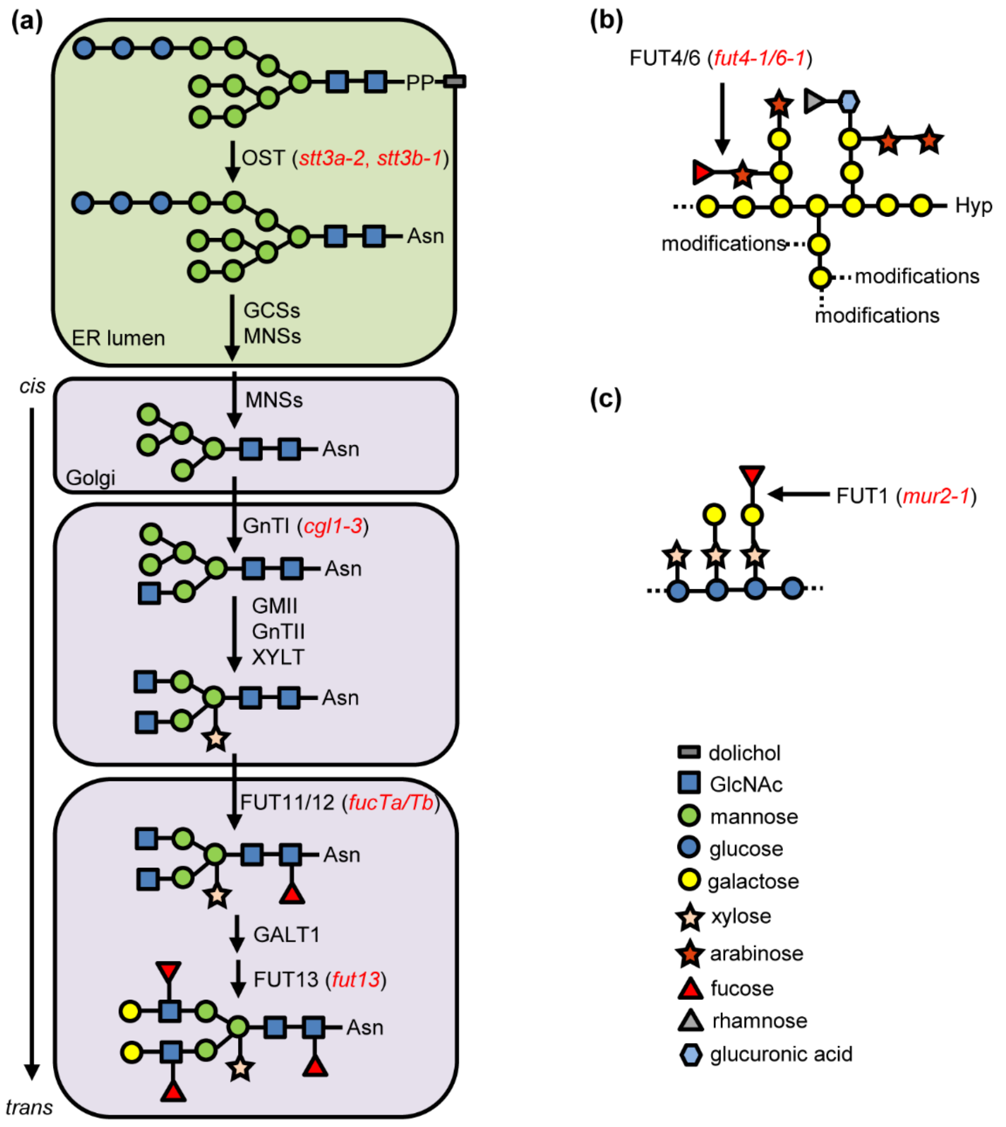

Fig. S9: Simplified diagrams of *N*-glycan processing and modification of *O*-glycan and xyloglucan.

(a) A simplified schematic diagram of *N*-glycan processing in the endoplasmic reticulum (ER) and Golgi (Strasser 2016). The OST complex catalyzes the transfer of a pre-assembled oligosaccharide from the lipid carrier dolichol pyrophosphate to a selected asparagine residue of the nascent polypeptide (Strasser 2016).

(b) A simplified schematic diagram of *O*-glycans attached to arabinogalactan proteins.

(c) A simplified schematic diagram of the XLFG (glucose/xylose/galactose/fucose) subunit of xyloglucan.

Arabidopsis mutants used for disease assays and stomatal assays are indicated in red with parentheses.

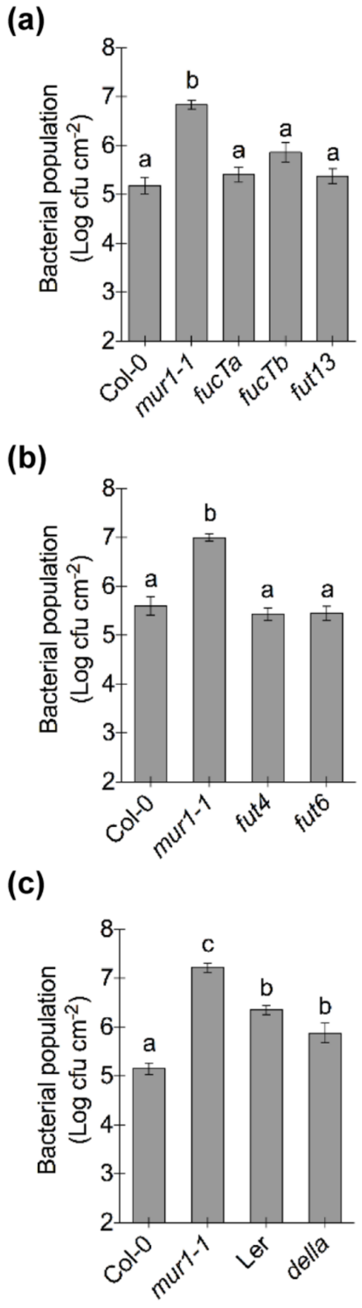

Fig. S10: Disease assays of Arabidopsis mutants of fucosyltransferases and quintuple *della* mutant.

Bacterial populations three days after dip-inoculation with 1×10^8 cfu ml^{-1} *Pst* DC3118 in fucosyltransferase single mutants for *N*-glycan (a) or *O*-glycan (b), or quintuple *della* mutant (c). Different letters above the columns indicate significant differences ($P < 0.05$) of bacterial

populations between genotypes by one-way ANOVA with Tukey's test ($n = 4$, error bars, $\pm$ SEM).

Table S1: List of primers.

name of primer	accession number	sequence	purpose	reference for primer	ABRC
SCORD6_FP<br>SCORD6_RP	AT3G51160	ATGGCGTCAGAGAACAACGGAT<br>AGGTTGCTGCTTAGCATCCATGTAT	*scord6* deletion confirmation		
SCORD6_BPF<br>SCORD6_BPR	AT3G51160	GGGGACAAGTTTGTACAAAAAAGCAGGCTTCATGGCGTCAGAGAACAACGGAT<br>GGGGACCACTTTGTACAAGAAAGCTGGGTCAGGTTGCTGCTTAGCATCCATGTAT	cloning *SCORD6* gene		
PP2AA3_qF<br>PP2AA3_qR	AT1G13320	GGTTACAAGACAAGGTTCACTC<br>CATTCAGGACCAAACTCTTCAG	qPCR	Zhang *et al*. 2015	
CYP81F2_qF<br>CYP81F2_qR	AT5G57220	GCCCGAGAAGTTTATGCCTGAG<br>CAACGAACCTAAAGCCAACAATACC	qPCR	J. Li *et al*. 2009	
FRK1_qF<br>FRK1_qR	AT2G19190	CATTAGATGCAGCGCAAGGAC<br>GGTTGGCCTGTAATCACTTC	qPCR		
mur1-1_F<br>mur1-1_R	AT3G51160	ATGGCGTCAGAGAACAACGGAT<br>AGGTTGCTGCTTAGCATCCATGTAT	genomic PCR		CS6243
mur2-1_F<br>mur2-1_R	AT2G03220	TCGGTGAGTGACTTTAGAGTCT<br>AATCATACTAGCTTAAGTCCCCA	genomic PCR		CS8565
fut4_F<br>fut4_R	AT2G15390	CCATGTAGTTACATTCCCAACCG<br>CCACGTCGATGGAGCCTTGTTT	genomic PCR	Liang *et al*. 2013	SAIL_284_B05
fut6_F<br>fut6_R	AT1G14080	CACATCTTTCAGATCTCCAGCG<br>CTTTCTTGTAAGCATCCGTGC	genomic PCR	Liang *et al*. 2013	SALK_099500
fucTa_F<br>fucTa_R	AT3G19280	TGCCACAACTTAGCATCTCCT<br>TAGGACCTCGAAGATTGGAGA	genomic PCR	Kaulfurst-Soboll *et al*. 2011	SALK_087481
fucTb_F<br>fucTb_R	AT1G49710	ATGAAGTATCTCGCAGCTAAC<br>AATGTGACTACTTAGACTCGA	genomic PCR	Kaulfurst-Soboll *et al*. 2011	SALK_063355
fut13-2_F<br>fut13-2_R	AT1G71990	ACATCGTCTTTCGCATGGTAG<br>GAGGATCTCCGGTGGTTTAAG	genomic PCR	Anderson *et al*. 2012	SALK_067444
cgl1-3_F<br>cgl1-3_R	AT4G38240	CGTGCAGACTATCTTG<br>CCATTATCATTCCATGATGAAGC	genomic PCR	Frank *et al*. 2008	SALK_073650
stt3a-2_F<br>stt3a-2_R	AT5G19690	ATTGCAAGTGTCAGTGAACATCAAC<br>CCTTGTCAGTCTTACCAGCAGAA	genomic PCR	Koiwa *et al*. 2003	SALK_058814
stt3b-1_F<br>stt3b-1_R	AT1G34130	ACTTGATGTGAACTATGTATTGGTCG<br>GGTTCTTTGATCACTGGGAATACTC	genomic PCR	Koiwa *et al*. 2003	SALK_033391
spy-3_F<br>spy-3_R	AT3G11540	ATCATTGGTCTCTCATGGCTT<br>TGTGACAAAGCCATTAGAAAGC	genomic PCR		CS6268
spy-5_F<br>spy-5_R	AT3G11540	TCAGTACATGCTCATAATGTTGGT<br>TTCCAGATGGGTCAAGACTC	genomic PCR		CS8094
LB3		TAGCATCTGAATTTCATAACCAATCTCGATACAC	T-DNA		

LBb1.3		ATTTTGCCGATTTCGGAAC	T-DNA		

Table S2: Summary of key assay results of the Arabidopsis mutants analyzed in this study

Mutant	Function of gene	Arabidopsis defense against *Pst* DC3118	Stomatal defense against *Pst* DC3118	Apoplastic defense against *Pst* DC3118	PTI	ETI
scord6, *mur1-1*	GDP-D-mannose-4,6-dehydratase	-	-	-	-	-
fucTa fucTb	α1,3-fucosyltransferases for N-glycan processing in Golgi	-	-	-		
fut13	α1,4-fucosyltransferase for N-glycan processing in Golgi	+				
stt3a-2	putative subunits of OST complex in ER	-	-	-		
stt3b-1	putative subunits of OST complex in ER	-	-	-		
cgl1-3	GnTI in Golgi	-	-	-		
fut4 fut6	arabinogalactan-protein specific α1,2-fucosyltransferase	-	+	-		
spy-3, *spy-5*	*O*-fucosyltransferase for mono-fucosylation of DELLAs	-	-*	-		
mur2-1	XyG specific α1,2-fucosyltransferase	+				
della	repressors of GA signaling	+				

+: Wild-type resistance

-: Compromised resistance

*: Only tested in the *spy-3* mutant

References

Anderson CT, Wallace IS, Somerville CR. 2012. Metabolic click-labeling with a fucose analog reveals pectin delivery, architecture, and dynamics in *Arabidopsis* cell walls. *Proceedings of the National Academy of Sciences of the United States of America* **109**: 1329-1334

Frank J, Kaulfurst-Soboll H, Rips S, Koiwa H, von Schaewen A. 2008. Comparative analyses of *Arabidopsis complex glycan1* mutants and genetic interaction with *staurosporin and temperature sensitive3a*. *Plant Physiology* **148**: 1354-1367

Kaulfurst-Soboll H, Rips S, Koiwa H, Kajiura H, Fujiyama K, von Schaewen. 2011. Reduced immunogenicity of *Arabidopsis hgl1* mutant *N*-glycans caused by altered accessibility of xylose and core fucose epitopes. *The Journal of Biological Chemistry* **286**: 22955-22964

Koiwa H, Li F, McCully MG, Mendoza I, Koizumi N, Manabe Y, Nakagawa Y, Zhu J, Rus A, Pardo JM, *et al.* 2003. The STT3a subunit isoform of the *Arabidopsis* oligosaccharyltransferase controls adaptive responses to salt/osmotic stress. *The Plant Cell* **15**: 2273-2284

Li J, Zhao-Hui C, Batoux M, Nekrasov V, Roux M, Chinchilla D, Zipfel C, Jones JD. 2009. Specific ER quality control components required for biogenesis of the plant innate immune receptor EFR. *Proceedings of the National Academy of Sciences of the United States of America* **106**: 15973-15978

Liang Y, Basu D, Pattathil S, Xu WL, Venetos A, Martin SL, Faik A, Hahn MG, Showalter AM. 2013. Biochemical and physiological characterization of *fut4* and *fut6* mutants defective in arabinogalactan-protein fucosylation in *Arabidopsis*. *Journal of Experimental Botany* **64**: 5537-5551

Strasser R. 2016. Plant protein glycosylation. *Glycobiology* **26**: 926-939

Zeng W, Brutus A, Kremer JM, Withers JC, Gao X, Jones AD, He SY. 2011. A genetic screen reveals *Arabidopsis* stomatal and/or apoplastic defenses against *Pseudomonas syringae* pv. *tomato* DC3000. *PLoS Pathogens* **7**: e1002291

Zhang L, Yao J, Withers J, Xin XF, Banerjee R, Fariduddin Q, Nakamura Y, Nomura K,

Howe GA, Boland W, *et al*. 2015. Host target modification as a strategy to counter pathogen hijacking of the jasmonate hormone receptor. *Proceedings of the National Academy of Sciences United States of America* **112**: 14354–14359
